# Quality assessment and Q-markers discovery of Tongsaimai tablet by integrating serum pharmacochemistry and network pharmacology for anti-atherosclerosis benefit

**DOI:** 10.1186/s13020-022-00658-9

**Published:** 2022-09-02

**Authors:** Yanfen Cheng, Meng Xiao, Jiamei Chen, Di Wang, Yichen Hu, Chenfeng Zhang, Tuanjie Wang, Chaomei Fu, Yihan Wu, Jinming Zhang

**Affiliations:** 1 State Key Laboratory of Southwestern Chinese Medicine Resources, School of Pharmacy, Chengdu University of Traditional Chinese Medicine, Chengdu, 611137 China; 2 Key Laboratory of Coarse Cereal Processing, Ministry of Agriculture and Rural Affairs, Chengdu University, Chengdu, 610106 Sichuan China; 3 Jiangsu Kanion Pharmaceutical CO. LTD, Lianyungang, 222001 China; 4 State Key Laboratory of New-Tech for Chinese Medicine Pharmaceutical Process, Lianyungang, 222001 China

**Keywords:** Tongsaimai tablet, Quality control, Serum pharmacochemistry, Network pharmacology, Atherosclerosis, Q-markers

## Abstract

**Background:**

The limited therapeutic outcomes of atherosclerosis (AS) have allowed, traditional Chinese medicine has been well established as an alternative approach in ameliorating AS and associated clinical syndromes. Clinically, Tongsaimai tablet (TSMT), a commercial Chinese patent medicine approved by CFDA, shows an obvious therapeutic effect on AS treatment. However, its effective mechanism and quality control still need thorough and urgent exploration.

**Methods:**

The mice were orally administered with TSMT and their serum was investigated for the absorbed compounds using serum pharmacochemistry via the UPLC-Q-Exactive Orbitrap/MS analysis was employed to investigate these absorbed compounds in serum of mice orally administrated with TSMT. Based on these absorbed prototype compounds in serum derived from TSMT, a component-target-disease network was constructed using network pharmacology strategy, which elucidated the potential bioactive components, effective targets, and molecular mechanisms of TSMT against AS. Further, the screened compounds from the component-target network were utilized as the quality control (QC) markers, determining multi-component content determination and HPLC fingerprint to assess quality of nine batches of TSMT samples.

**Results:**

A total of 164 individual components were identified in TSMT. Among them, 29 prototype compounds were found in serum of mice administrated with TSMT. Based on these candidate prototype components, 34 protein targets and 151 pathways related to AS were predicted, and they might significantly exhibit potential anti-AS mechanisms via synergistic regulations of lipid regulation, shear stress, and anti-inflammation, etc. Five potentially bioactive ingredients in TSMT, including Ferulic acid, Liquiritin, Senkyunolide I, Luteolin and Glycyrrhizic acid in quantity not less than 1.2798, 0.4716, 0.5419, 0.1349, 4.0386 mg/g, respectively, screened from the component-target-pathway network. Thereby, these indicated that these five compounds of TMST which played vital roles in the attenuation of AS could serve as crucial marker compounds for quality control.

**Conclusions:**

Overall, based on the combination of serum pharmacochemistry and network pharmacology, the present study firstly provided a useful strategy to establish a quality assessment approach for TSMT by screening out the potential anti-AS mechanisms and chemical quality markers.

**Graphical Abstract:**

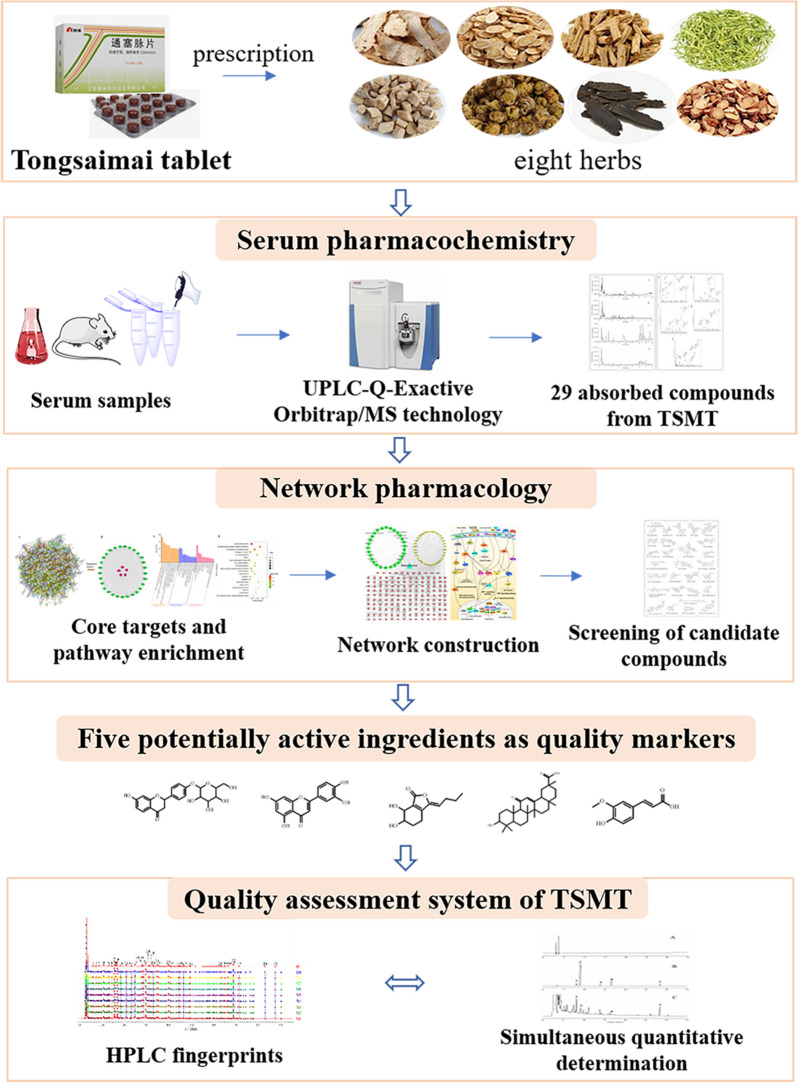

**Supplementary Information:**

The online version contains supplementary material available at 10.1186/s13020-022-00658-9.

## Introduction

Atherosclerosis (AS) is presented as the main pathological process in the heart, brain, and peripheral vascular diseases. It is characterized by the accumulation of pathological plaque and inflammation of the arterial wall [1]. AS also remains the leading cause of morbidity and mortality worldwide and may induce coronary heart disease, thrombosis, stroke, cerebrovascular infarction and other related serious complications [2]. Due to the insufficient therapeutic outcomes and potential side effects of commercial medicines such as statins [3], traditional Chinese medicine (TCM) are being increasingly recommended for the prevention and treatment of AS. Some Chinese patent medicines, such as Xue Zhi Kang Capsule and Shan Zha Jian Zhi tablet along with their bioactive herbal components, namely Ginkgolides and tanshinones, have exhibited significant efficacy in clinical or pre-clinical studies [4, 5].

Based on TCM theory, Tongsaimai tablet (TSMT), a commercial Chinese patent medicine, exhibits the medicinal function of promoting blood circulation, dredging collaterals, tonifying Qi, and nourishing Yin [6]. TSMT has been commonly applied to improve these clinical symptoms associated with AS, including regulation of inflammation, stabilization of plaque, protection of vascular endothelial cells, regulation of blood lipid, inhibition of blood coagulation, and so on [7–9]. Furthermore, the herbs in the prescription of TSMT, namely *Angelica sinensis* (Oliv.) Diels, *Lonicera japonica* Thunb., *Codonopsis pilosula* (Franch.) Nannf., *Scrophularia aestivalis* Griseb, *Astragalus membranaceus* (Fisch.) Bunge, *Achyranthes bidentata* Bl., *Dendrobium nobile* Lindl., and *Glycyrrhiza uralensis* Fisch., ameliorated AS or peripheral vascular diseases. However, only a few studies have investigated the mechanisms of TSMT in ameliorating AS. Moreover, despite the affirmed clinical benefits of TSMT, its quality control and assessment remain greatly limited. Based on the mandatory quality control standards, the only the recommended quantitative determination was of chlorogenic acid, which was mainly derived from *Lonicera japonica* Thunb. [10]. The holistic view of TCM theory believes that traditional Chinese medicinal herbs work via the complex interactions among the complex disease targets and a variety of chemical constituents [11]. However, exploring the ingredients responsible for therapeutic efficacy remains a matter of concern. Unfortunately, the current quality control index of chlorogenic acid cannot represent the effective material basis for TSMT. Therefore, there is a need to promote rational quality assessment of TSMT based on the real quality control markers.

**Fig. 1:**
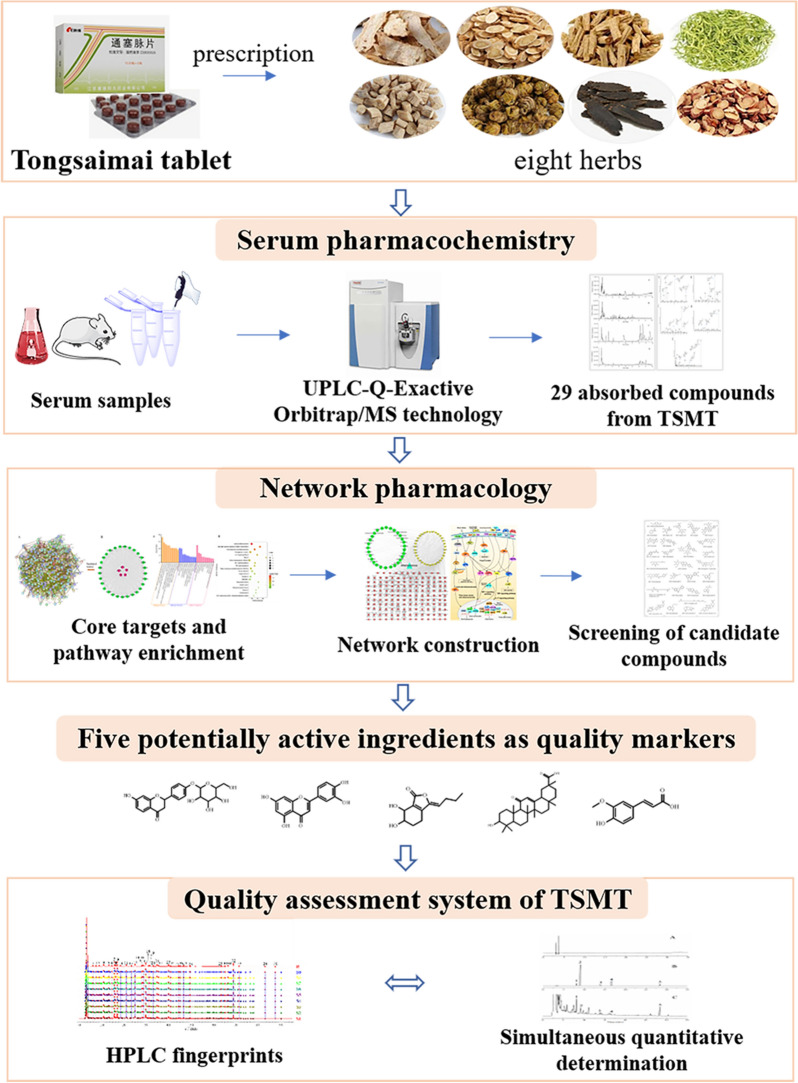
Schematic diagram exploring the quality assessment of Tongsaimai tablets by integrating serum pharmacochemistry and network pharmacology

Since it is a well-acknowledged viewpoint that only the components absorbed into serum are the potential bioactive material basis of medicinal herbs [12], we used serum pharmacochemistry to analyze the prototype phytochemical compounds in TSMT prescription. As a proof of concept, the absorbed phytochemical constituents of TSMT in mice serum were identified using a UPLC-Q-Exactive Orbitrap/MS analysis approach. Next, serum pharmacochemistry integrated with network pharmacology [11] was used to examine the potentially active components in TSMT and their possible mechanisms ameliorating AS. This approach has also been corroborated as a useful approach in our previous study [13]. Based on the predicted effective phytochemicals in TSMT, we developed both the quantitative determination of multiple components by HPLC and chromatographic fingerprint to assess the quality of multiple batches of TSMT samples. To the best of our knowledge, this is the first report to explore the quality control aspect of TSMT based on the potential effective compounds and their therapeutic benefits. Figure 1 depicts the schematic representation of the study.

## Materials and methods

### Materials and chemicals

We obtained the nine batches of TSMT samples (S1-S9, No. 210401 ~ 210409) from Jiangsu Kanion Pharmaceutical Co., Ltd (Jiangsu, China). These were prepared from eight herbal pieces, i.e., *Angelica sinensis* (Oliv.) Diels (DG, danggui), *Lonicera japonica* Thunb. (JYH, jinyinhua), *Codonopsis pilosula* (Franch.) Nannf. (DS, dangshen), *Scrophularia aestivalis* Griseb (XS, xuanshen), *Astragalus membranaceus* (Fisch.) Bunge (HQ, huangqi), *Achyranthes bidentata* Bl. (NX, niuxi), *Dendrobium nobile* Lindl. (SH, shihu), and *Glycyrrhiza uralensis* Fisch. (GC, gancao) which were also checked with http://www.theplantlist.org on February 14, 2022. Next, we purchased 17 reference standards (RS) from Chengdu MUST Bio-Technology Co, Ltd (Chengdu, China), which included Astragaloside IV, Calycosin-7-glucoside, Atractylenolide II, Atractylenolide III, Ferulic acid, Senkyunolide I, Cynaroside, Chlorogenic acid, Luteolin, Harpagoside, Harpgide, Acteoside, *β*-Ecdysone, Ginsenoside Ro, Glycyrrhizic acid, Liquiritin, and Gallic acid. The purity of all the reference standards was over 98%. Mobile phases including the HPLC-grade acetonitrile, phosphoric acid, and formic acid were purchased from Thermo Fisher Scientific (Thermo scientific, USA).

### Preparation of standards and TSMT samples

For qualitative identification of the chemicals, all reference standards were dissolved in methanol for the UPLC-Q-Exactive Orbitrap/MS analysis. Additionally, for HPLC fingerprint and content determination analysis, the mixed reference solution was prepared including five standards including Ferulic acid, Liquiritin, Senkyunolide I, Luteolin, and Glycyrrhizic acid, at the concentration of 84.60, 360.00, 132.40, 112.40, 221.60 mg/L, respectively. It was generated six different concentration gradients for HPLC fingerprint and content determination analysis. All standard solutions were stored at 4 °C before use.

Approximately 0.35 g of TSMT samples were fully ground, and diluted in 10 mL of 70% methanol (v/v) for 45 min using ultrasonication. Finally, the TSMT samples from each batch were filtered through a 0.22 µm membrane filter after centrifugation at 10,000 rpm for 10 min. All TSMT sample solutions were stored at 4 °C before performing UPLC-QTOF-MS analysis, followed by determination and fingerprint analysis.

### Preparation of serum samples from mice administrated with TSMT

Male BALB/c mice (aged 4–6 weeks and weighting 16 ~ 18 g, certificate number: SCXK (Beijing) 2019–0010) were obtained from SPF Biotechnology Co., Ltd. (Beijing, China). All animals were kept at 12 h light/dark cycle at the animal care facility under standard conditions. The animals were acclimatized for at least 7 days before the start of the experiment, and were fed a fresh diet with free access to water. The animal experiments were approved by the ethics committee of the Chengdu University of Traditional Chinese Medicine (CDUTCM, permit CDU2020KB097), and were conducted in strict accordance with the Guidelines for the Care and Use of Laboratory Animals of the Ministry of Science and Technology of China.

For serum pharmacochemistry study, mice were orally administered with 0.6825 g/kg of TSMT solution, while sterile saline was used as the blank group. After 1 h of oral administration via the retroorbital sinus, blood samples (500 µL) were collected and centrifuged at 3500 rpm for 15 min. Next, 600 µL of acetonitrile was added to 200 µL of serum samples, vortexed, and centrifuged at 12,000 rpm for 10 min for protein-precipitation. The obtained supernatant was dried using nitrogen gas. The precipitate was redissolved in a 1 mL mobile phase, followed by swirling and centrifugation at 12,000 rpm for 15 min. Finally, the collected supernatant was used to perform UPLC-Q-Exactive Orbitrap/MS analysis.

### UPLC-Q-exactive orbitrap/MS analysis

Dionex UltiMate 3000 Rapid Separation UPLC system (Thermo Fisher Scientific Inc., USA) equipped with a DAD-3000RS detector was used to carry out the chromatographic analysis. The chromatographic separation was performed on an Accucore™ C18 column (100 × 2.1 mm, 2.6 µm, Thermo Fisher Scientific Inc., USA) at 30 °C. The mobile phases were composed of 0.1% formic acid in water (A) and acetonitrile (B) (v/v) with a flow rate of 0.30 mL/min and an injection volume of 2 µL. The gradient elution program was as follows: 0 ~ 10 min 5 ~ 20% B, 10 ~ 25 min 20% ~ 60% B, 25 ~ 40 min 60% ~ 95% B. The online UV spectra were recorded in the range of 190 ~ 400 nm.

To identify chemical compounds in both TSMT samples and serum samples of mice administered with TSMT, we used ultra-performance liquid chromatography Q-Exactive Orbitrap tandem mass spectrometry (UPLC-Q-Exactive Orbitrap/MS) (Thermo Fisher Scientific Inc., USA) fitted with electrospray ionization (ESI) source, where the following experimental parameters setting were used: ion spray voltage, 3 kV; collision energy, 35 eV; capillary voltage,4 kV; capillary temperature, 320 °C; heater temperature, 300 °C; sheath gas flow rate, 35 Arb; auxiliary gas flow rate, 10 Arb. Both positive and negative ion modes were performed on the instrument with the full scan range of 50–1500 m/z. Data were processed and analyzed using Xcalibur™ version2.2.1 and Trace Finder3.3 version (Thermo Fisher Scientific Inc, USA) along with compound discoverer 3.0 software (Thermo Fisher Scientific Inc., USA). Molecular qualitative analysis of all samples was measured in contrast with retention times and MS spectra through available reference standards.

### Network pharmacology analysis

The structural formulas (*.sdf / SMILES) of potential active components were identified using UPLC-Q-Exactive Orbitrap/MS, which were determined and downloaded from ChemSpider (http://www.chemspider.com/) and PubChem (https://pubchem.ncbi.nlm.nih.gov/) and drawn using Chemdraw 18.0 software. Next, all predicted targets of components were obtained from Swiss Target Prediction (http://www.swisstargetprediction.ch/) to perform reverse docking based on structural similarity. Additionally, GeneCards (https://www.genecards.org/) was used to obtain the targets closely related to AS. All the target names were standardized into official gene symbols (Homo sapiens) using the UniProt database (http://www.uniprot.org/). Finally, the intersecting targets overlapping the TSMT compound targets and AS-related genes were obtained.

To filter out and select functional core targets, we constructed the protein–protein interaction (PPI, *p*-value < 0.01) for the intersection targets between TSMT compounds and AS disease using the STRING database (https://string-db.org/), where “homo sapiens” species was selected. The PPI network from the STRING database was then imported into Cytoscape 3.7.1 software to construct target network. Meanwhile, the analyzer plugin of Cytoscape 3.7.1 was used to calculate all nodes with the topological property of Betweenness, Closeness, and degree centrality representing predicted targets in the network. Finally, the candidate hub targets were screened at degree values higher than 99.

To further characterize the functional meaning and explain the biological roles of the above confirmed key targets, the Discovery database (DAVID 6.8, https://david.ncifcrf.gov/home.jsp) was used for performing Gene ontology (GO) analysis including biological processes, cellular components, and molecular function. Further, to perform target annotation, visualization, and integration (*P-*value < 0.01), the enrichment pathway analysis was carried out based on Kyoto Encyclopedia of Genes and Genomes database (KEGG, http://www.genome.jp/kegg/). As a result, a “component-targets-pathway-disease” network was constructed by means of Cytoscape 3.7.1 software to reveal the underlying mechanism of the disease. Moreover, Additionally, R 4.0.3 software was used to draw the bubble map to visualize the results of the pathway annotation. Therefore, initially, we selected the above active compositions related closely to AS targets as candidates for establishing a quality assessment system for TSMT.

### Determination of five markers of TSMT

#### HPLC instrumentation and conditions

Quantitative analysis and fingerprint analysis were performed using UltiMate 3000 HPLC instrument (Thermo-Fisher, USA) with a DAD 3000 detector, a ternary pump of SR3000 Solvent Rack, a WPS-3000SL autosampler, a TCC-3000SD column temperature controller, and a workstation of Chromeleon 7.2. The compounds separation was performed on a Thermo Hypersil Gold C18 column (250 mm × 4.6 mm, 5 μm). The mobile phase consisted of 0.1% phosphoric acid (solvent A) and Acetonitrile (solvent B) at a flow rate of 1 mL/min, which followed a gradient program of 0–20 min, 18–35% B; 20–30 min, 35–48% B; 30–35 min. The column temperature was maintained at 30 °C while the autosampler temperature was maintained at 4 °C with 10 μL of sample solution being injected. Both the detection wavelength of DAD was monitored at 235 nm.

#### Validation of the method

According to the recommendations and guidelines of *Chinese Pharmacopoeia*, the validation of the HPLC content determination method included calibration curves, precision, stability, repeatability, and recovery tests. Among these, we established the calibration curves by analyzing the mixed standard solutions containing five reference substances at six concentration levels. These curves were drawn by plotting the relationship between different peak areas (y) and the corresponding concentration (x, mg/L) of analytes with least-square linear regression to conduct calibration curves. For confirmatory methods, the correlation coefficient (*r*) of linear regression equations should not be lower than 0.999. The precision was measured by conducting six consecutive analyses of one sample solution. The repeatability was assessed by analyzing six parallel sample solutions prepared from the same batch. The stability was estimated using replicates of one sample solution at different time points (0, 2, 4, 8, 12, 24 h). To determine the precision, stability, and repeatability of the test solution, Relative Standard Deviation (RSD%) was calculated. Finally, the recovery test, i.e., adding reference standards of corresponding compounds at certain levels into the above sample solutions, was performed by analyzing six parallel solutions from the same batch. Thus, the accuracy of the method was confirmed. Additionally, to evaluate the average recovery of analytes, the corresponding peak areas were measured and analyzed.

#### Determination of the contents

Sample solutions for five potentially effective compounds in nine batches of TSMT were prepared using the above-mentioned method. Of which, 10 µL was injected under HPLC conditions to measure the peak area of each compound and calculate all TSMT contents. However, the content determination limit needs further verification to exhibit better control over the quality of the TSMT preparation.

### HPLC fingerprint analysis

#### HPLC conditions

The chromatographic conditions for fingerprint analysis were the same as mentioned for quantitative analysis, except, the gradient elution for the chemical fingerprint was as follows: 0–25 min, 5–17% B; 25–50 min, 17–25% B; 50–80 min, 25–55% B; 80–95 min, 55–95%.

#### Validation of the methods

The validation of fingerprint method including repeatability, precision, and stability tests were also measured using the above-mentioned method. A reference peak was set as per the chemical profiles and characteristics of fingerprints, after which relative peak areas (RPA) of common peaks (the peak areas > 0.2) were used to detect and evaluate the fingerprint. RSD% was calculated and evaluated the results of RPA for the precision, stability, and repeatability of the test solution.

#### Similarity analysis

Based on the fingerprint chromatographic conditions for the fingerprint method, each chromatogram file of nine detected samples of TSMT was derived with “*.cdf” format using Chromeleon 7.2 workstation. It was then manipulated by a professional software called Similarity Evaluation System for Chromatographic Fingerprint of Traditional Chinese Medicine, provided by the Chinese Pharmacopoeia Committee (Version 2012A) (Beijing, China). This calculated and generated simulated reference fingerprints, while separately counted separately the correlation coefficients of similarity between nine batches of TSMT and simulated reference fingerprints.

## Results

### Identification of absorbable TSMT compounds

**Fig. 2:**
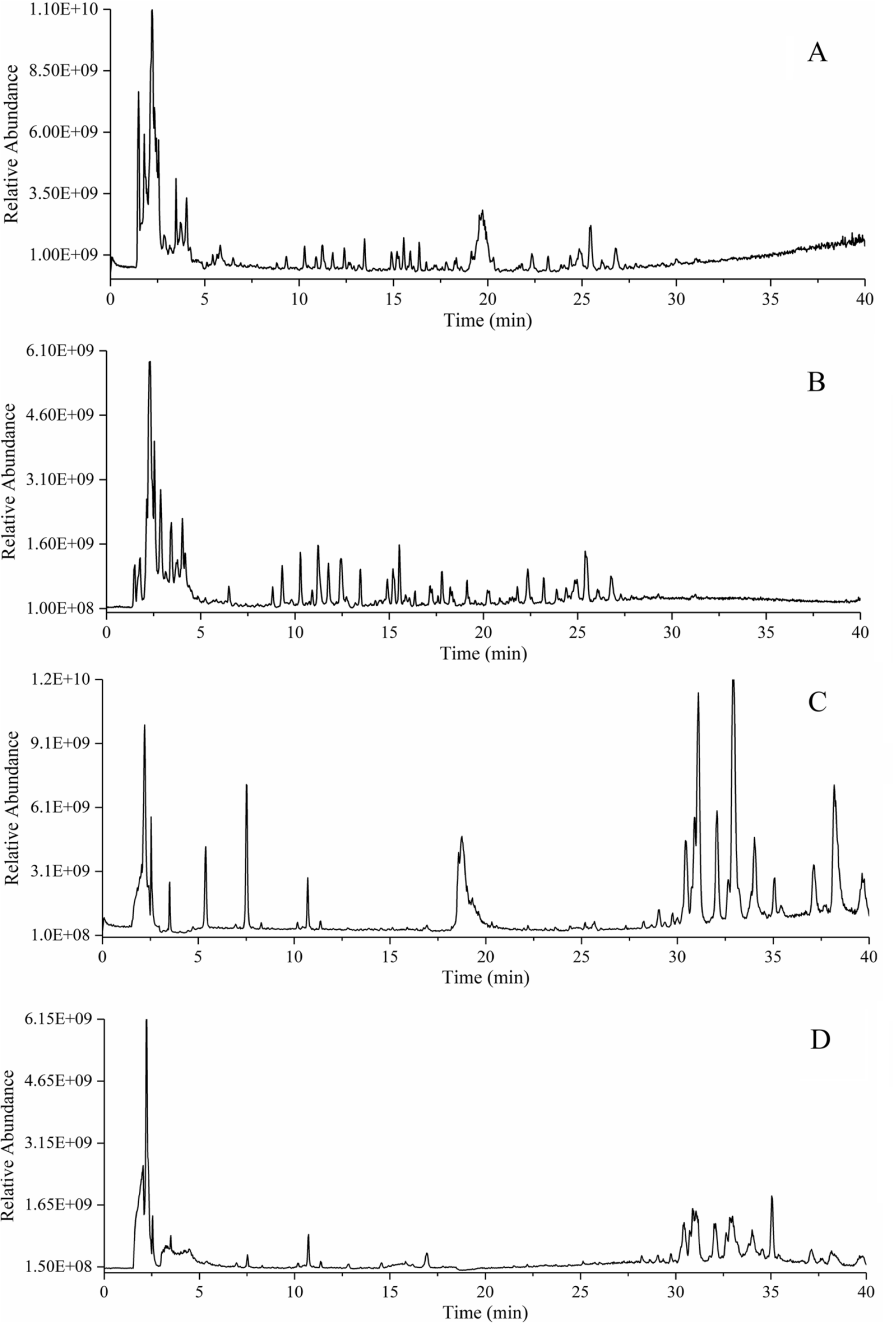
Base peak chromatograms of TSMT samples by UPLC-Q-Exactive Orbitrap/MS in positive (**A**) and negative modes (**B**), and base peak chromatograms of TSMT-derived serum in positive (**C**) and negative modes (**D**)

Both TSMT extracted sample and serum samples from mice administrated with TSMT were analyzed using UPLC-Q-Exactive Orbitrap/MS with positive and negative ion mode. The current total ion chromatograms of TSMT and serum sample were presented in Fig. 2. Based on the reference standards, and related studies comparing with the accurate mass and retention time information of compounds, we then characterized 164 chemical components from TSMT extraction in vitro. All detailed information was shown in Table 1.

**Table 1:** Identification results of all constituents of TSMT in vitro and in vivo by UPLC-Q-exactive MS/MS via MS data in (+ / −) ESI modes

Peak no	tR/min	Name	Formula	Mass ion type	Mean measured	Theoretical exact	Mass error(ppm)	Fragment ions	References
1	2.28	5-Hydroxymethylfurfural ^M^	C_6_H_6_O_3_	[M + H]^+^	127.0389	127.0390	− 0.79	127.0389, 109.0286, 81.0340	[15, 37]
2	2.39	Achyranthine ^M^	C_6_H_11_NO_2_	[M + H]^+^	130.0862	130.0863	− 0.77	130.0862, 112.0760, 84.0812	[37]
3	2.90	Quinic acid ^M^	C_7_H_12_O_6_	[M−H]^−^	191.0555	191.0561	− 3.14	191.0555, 173.0444, 127.0391, 85.0284	[38]
4	3.32	Codonopsinol A	C_13_H_19_NO_5_	[M + H]^+^	270.1331	270.1336	− 1.85	270.1331, 74.0606	[39]
5	3.42	Codonopsinol	C_14_H_21_NO_5_	[M + H]^+^	284.1488	284.1492	− 1.41	284.1488, 88.0761	[39]
6	3.63	codonopiloside A	C_19_H_29_NO_9_	[M + H]^+^	416.1917	416.1915	0.48	416.1917, 254.1384, 236.1097, 187.0753, 161.0597	[39]
7	4.03	Codonopsinol B	C_13_H_19_NO_4_	[M + H]^+^	254.1387	254.1387	0.00	254.1387, 236.1286, 161.0594	[39]
8	4.24	Codonopsine	C_14_H_21_NO_4_	[M + H]^+^	268.1541	268.1543	− 0.75	268.1541, 88.0761	[40]
9	5.71	Gallic acid ^#^	C_7_H_6_O_5_	[M−H]^−^	169.0134	169.0142	− 4.73	169.0134, 125.0235	[38]
10	6.49	Harpagide ^#^	C_15_H_24_O_10_	[M−H]^−^	363.1292	363.1297	− 1.38	363.1292, 201.0763, 183.0656, 165.0548, 139.0931	[41]
11	6.92	3,4-dihydroaucubin	C_15_H_24_O_9_	[M + COOH]–	393.1398	393.1402	− 1.02	393.1398, 347.1346, 239.4955, 167.0707,127.0392	[42]
12	7.10	Woodorien	C_14_H_18_O_9_	[M−H]^−^	329.0890	329.0878	3.65	329.0890, 167.0342, 152.0107, 123.0441, 108.0207	[40]
13	7.61	Danshensu	C_9_H_10_O_5_	[M−H]^−^	197.0448	197.0455	− 3.55	197.0448, 179.0342, 152.9037, 135.0442, 123.0442	[43]
14	7.73	Vanilloside	C_14_H_18_O_8_	[M + COOH]−	359.0985	359.0984	0.28	359.0985, 313.0928, 197.0449, 161.0448, 151.0392	[44]
15	8.09	Decaffeoylacteoside	C_20_H_30_O_12_	[M−H]^−^	461.1667	461.1664	0.65	461.1667, 315.1097, 161.0446, 135.0443,113.0235	[41]
16	8.12	8-epi-Loganic acid	C_16_H_24_O_10_	[M−H]^−^	375.1292	375.1297	− 1.33	375.1290, 213.0763, 169.0863, 151.0760, 113.0235	[38]
17	8.86	Loganic acid ^M^	C_16_H_24_O_10_	[M−H]^−^	375.1290	375.1297	− 1.87	375.1290, 213.0763, 169.0863, 151.0760, 113.0235	[38]
18	8.88	Isoferulic acid	C_10_H_10_O_4_	[M + H]^+^	195.0647	195.0652	− 2.56	195.0647, 177.0545, 149.0596	[15, 39, 43, 45]
19	9.24	Caffeic acid	C_9_H_8_O_4_	[M + H]^+^	181.0499	181.0495	2.21	181.0499, 163.0388, 135.0440, 89.0390	[46, 47]
20	9.31	Chlorogenic acid isomer	C_16_H_18_O_9_	[M−H]^−^	353.0878	353.0878	0.00	353.0878, 191.0555, 179.0343, 161.0235, 135.0443	[47]
21	10.09	Tangshenoside II	C_17_H_24_O_9_	[M−H]^−^	371.1343	371.1348	− 1.35	371.1343, 209.0645, 191.0906	[40]
22	10.10	Methyl caffeate	C_10_H_10_O_4_	[M + H]^+^	195.0652	195.0652	0.00	195.0652, 163.0656, 133.0284	[38]
23	10.21	Quercetin di-O-glucoside	C_27_H_30_O_17_	[M−H]^−^	625.1404	625.1410	− 0.96	625.1404, 463.0866, 299.0197, 151.0029	[42]
24	10.29	Aucubin	C15H22O9	[M−H]^−^	345.1191	345.1191	0.00	345.1191, 183.0659, 165.0550, 139.0753, 121.0649	[41]
25	10.31	Secologanoside	C_16_H_22_O_11_	[M−H]^−^	389.1086	389.1089	− 0.77	389.1086, 345.1182, 209.0451, 183.0658,165.0547, 121.0649	[41]
26	10.74	Secologanic acid or isomer	C_16_H_22_O_10_	[M−H]^−^	373.1143	373.1140	0.80	373.1143, 211.0606, 193.0500, 149.0596, 123.0446	[41]
27	10.96	Liquiritigenin 4',7-diglucoside isomer	C_27_H_32_O_14_	[M−H]^−^	579.1699	579.1719	− 3.45	579.1699, 255.0660, 153.0184, 119.0492	[41]
28	11.23	Chlorogenic acid ^#^	C_16_H_18_O_9_	[M−H]^−^	353.0878	353.0878	0.00	353.0878, 191.0555, 179.0343, 163.0388, 161.0235, 135.0440	[47]
29	11.59	Cistanoside F	C_21_H_28_O_13_	[M−H]^−^	487.1453	487.1457	− 0.82	487.1453, 307.0816, 163.0392, 145.0287	[48]
30	11.68	Loganin ^M^	C_17_H_26_O_10_	[M−H]^−^	435.1505	435.1508	− 0.69	435.1505, 227.0923, 210.0761, 127.0391, 101.0234	[41]
31	11.77	Neochlorogenic acid	C_16_H_18_O_9_	[M−H]^−^	353.0877	353.0878	− 0.28	353.0877, 191.0555, 179.0448, 161.0236, 135.0442	[47]
32	12.05	Vicenin-2 ^M^	C_27_H_30_O_15_	[M−H]^−^	593.1499	593.1512	− 2.19	593.1499, 503.1164, 473.1091, 383.0769, 353.0666	[45]
33	12.06	3-Feruloylquinic acid	C_17_H_20_O_9_	[M−H]^−^	367.1038	367.1035	0.82	367.1038, 193.0500, 173.0444, 134.0364	[41]
34	12.40	Sweroside ^M^	C_16_H_22_O_9_	[M + H]^+^	359.1329	359.1337	− 2.23	359.1329, 197.0807, 127.0390	[41]
35	12.76	Aucubin isomer	C_15_H_22_O_9_	[M−H]^−^	345.1187	345.1191	− 1.16	345.1187, 183.0657, 165.0549, 139.0757, 121.0648	[41]
36	13.28	Schaftoside	C_26_H_28_O_14_	[M−H]^−^	563.1401	563.1406	− 0.89	563.1401, 545.1277, 503.1202, 473.1082, 443.0988, 353.0663	[14, 18, 45]
37	13.44	Benzoic acid	C_7_H_6_O_2_	[M + H]^+^	123.0442	123.0441	0.81	123.0442, 95.0495, 79.0184	[15]
38	13.48	Kingiside ^M^	C_17_H_24_O_11_	[M−H]^−^	403.1250	403.1246	0.99	403.1250, 371.0981, 223.0604, 179.0555, 165.0550, 121.0285	[41]
39	13.79	Schaftoside isomer	C_26_H_28_O_14_	[M−H]^−^	563.1401	563.1406	− 0.89	563.1401, 503.1207, 473.1065, 443.0972, 353.0665	[14, 18, 45]
40	14.02	Caffeic acid hexoside	C_16_H_22_O_8_	[M−H]^−^	341.1238	341.1242	− 1.17	341.1238, 179.0708, 161.0600	[38]
41	14.26	(E)-2-Hexenyl-a-L arabinopyranosyl- (1/2)-b-D glucopyranoside	C_17_H_30_O_10_	[M−H]^−^	393.1756	393.1766	− 2.54	393.1756, 261.1332, 179.0554, 149.0448	[40]
42	14.33	5-Feruloylquinic acid	C_17_H_20_O_9_	[M−H]^−^	367.1022	367.1035	− 3.54	367.1022, 193.0554, 173.0447, 134.0363	[41]
43	14.44	Isoviolanthin	C_27_H_30_O_14_	[M−H]^−^	577.1556	577.1563	− 1.21	577.1556, 269.0454	[45]
44	14.61	6-O-Methylaucubin	C_16_H_24_O_9_	[M−H]^−^	359.1342	359.1348	− 1.67	359.1342, 197.0813, 153.0911	[42]
45	14.71	Apigenin-6,8-di-C-*β*-D-xyloside	C_25_H_26_O_13_	[M−H]^−^	533.1293	533.1301	− 1.50	533.1293, 515.1181, 473.1078, 443.0986, 383.0771, 353.0606, 297.0768, 135.0442	[49]
46	14.79	Hexyl *β*-sophoroside	C_18_H_34_O_11_	[M−H]^−^	425.2025	425.2028	− 0.71	425.2025, 263.1495, 161.0447	[50]
47	14.85	*β*-Ecdysone^#^	C_27_H_44_O_7_	[M + H]^+^	481.3151	481.3160	− 1.87	481.3151, 445.2942, 371.2211, 165.1273	[38]
48	14.91	Rutin	C_27_H_30_O_16_	[M−H]^−^	609.1454	609.1461	− 1.15	609.1456, 300.0272, 271.0246, 255.0295, 243.0294, 151.0028	[38, 45]
49	14.94	Calycosin-7-O-*β*-D-glucoside^#^	C_22_H_22_O_10_	[M + H]^+^	447.1287	447.1286	0.22	447.1287, 283.0610, 268.0375, 239.0346, 211.0394	[50]
50	14.94	Lonicerin	C_27_H_30_O_15_	[M−H]^−^	593.1506	593.1512	− 1.01	593.1506, 285.0403	[39]
51	14.98	20,26-dihydroxyecdysone	C_27_H_44_O_8_	[M + COOH]−	541.3009	541.3018	− 1.66	541.3009, 495.2962, 477.2855, 335.1869	[37]
52	15.12	Vitexin	C_21_H_20_O_10_	[M + H]^+^	433.1124	433.1129	− 1.15	433.1124, 313.0701, 283.0599	[45]
53	15.18	Acteoside ^#^	C_29_H_36_O_15_	[M−H]^−^	623.1976	623.1981	− 0.80	623.1976, 461.1656, 315.1098, 179.0345, 161.0236, 113.0286	[41]
54	15.23	Isoliquiritin	C_21_H_22_O_9_	[M−H]^−^	417.1188	417.1191	− 0.72	417.1188, 255.0660, 135.0078, 119.0493	[18]
55	15.24	Liquiritigenin ^M^	C_15_H_12_O_4_	[M + H]^+^	257.0804	257.0808	− 1.56	257.0804, 239.0702, 147.0439, 137.0232	[18]
56	15.25	Isoliquiritin apioside	C_26_H_30_O_13_	[M−H]^−^	549.1608	549.1614	− 1.09	549.1608, 255.0660, 135.0078, 119.0492	[41]
57	15.27	Nicotiflorin or isomer ^M^	C_27_H_30_O_15_	[M−H]^−^	593.1507	593.1512	− 0.84	593.1507, 447.0921, 285.0403, 151.0029	[41]
58	15.31	Liquiritigenin 4',7-diglucoside	C_27_H_32_O_14_	[M−H]^−^	579.1699	579.1719	− 3.45	579.1699, 255.0660, 153.0184, 119.0492	[41]
59	15.31	5-Hydroxylliquiritin	C_21_H_22_O_10_	[M−H]^−^	433.1130	433.1140	− 2.31	433.1130, 271.0610, 151.00328	[14]
60	15.32	Naringenin or isomer	C_15_H_12_O_5_	[M + H]^+^	273.0753	273.0757	− 1.46	273.0753, 153.0181, 147.0439, 119.0492	[41, 44]
61	15.42	Senkyunolide H	C_12_H_16_O_4_	[M + H]^+^	225.1131	225.1121	4.44	225.1131, 207.1014, 165.0909, 161.0958	[16]
62	15.52	Tangshenoside V isomer	C_21_H_26_O_12_	[M−H]^−^	469.1346	469.1351	− 1.07	469.1336, 325.0927, 265.0715, 235.0607, 163.0393, 19.0493	[50]
63	15.58	Cynaroside ^#^	C_21_H_20_O_11_	[M−H]^−^	447.0929	447.0933	− 0.89	447.0929, 285.0403	[38]
64	15.58	Quercetin	C_15_H_10_O_7_	[M + H]^+^	303.0494	303.0499	− 1.65	303.0494, 285.0393, 257.0441, 229.0495, 153.0181	[46]
65	15.58	Hyperoside	C_21_H_20_O_12_	[M−H]^−^	463.0880	463.0882	− 0.43	463.0880, 300.0273, 271.0247, 255.0297	[41]
66	15.60	Lobetyolinin	C_26_H_38_O_13_	[M + COOH]−	603.2290	603.2294	− 0.66	603.2290, 323.0977, 221.0661, 179.0554	[39]
67	15.60	p-Coumaric acid ^M^	C_9_H_8_O_3_	[M−H]^−^	163.0394	163.0401	− 4.29	163.0394, 119.0494	[41, 44, 50]
68	15.63	Liquiritin ^M^	C_21_H_22_O_9_	[M−H]^−^	417.1187	417.1191	− 0.96	417.1187, 255.0660, 135.0078, 119.0493, 91.0179	[18]
69	15.74	Sibirioside A	C_21_H_28_O_12_	[M + COOH]−	517.1558	517.1563	− 0.97	517.1558,471.1507, 323.0979, 189.0550, 161.0598, 147.0443	[41]
70	15.84	Isoacteoside	C_29_H_36_O_15_	[M−H]^−^	623.1978	623.1981	− 0.48	623.1978, 461.1673, 315.1076, 179.0343, 161.0236	[41]
71	15.94	Hexyl-(pen)-glucoside	C_17_H_32_O_10_	[M + COOH]−	441.1977	441.1978	− 0.23	441.1977, 395. 1919, 263.1497, 161.0446	[50]
72	16.02	Vallinic acid	C_8_H_8_O_4_	[M−H]^−^	167.0342	167.0350	− 4.79	167.0342, 123.0442	[14, 40, 41, 45, 46]
73	16.06	Naringin	C_27_H_32_O_14_	[M−H]^−^	579.1722	579.1719	0.52	579.1722, 417.1551, 387.1083, 181.0499, 166.0264	[49]
74	16.28	Angoroside C	C_36_H_48_O_19_	[M−H]^−^	783.2706	783.2717	− 1.40	783.2701, 607.2206, 193.0507, 175.0393, 160.0158	[41]
75	16.32	Violanthin	C_27_H_30_O_14_	[M−H]^−^	577.1558	577.1563	− 0.87	577.1558, 503.1201, 457.1105, 383.0772	[45]
76	16.33	Morroniside	C_17_H_26_O_11_	[M−H]^−^	405.1403	405.1402	0.25	405.1403, 243.1232, 225.1115, 101.0235	[41]
77	16.39	Ferulic acid ^# M^	C_10_H_10_O_4_	[M + H]^+^	195.0660	195.0652	4.10	195.0660, 177.0544, 149.0595, 145.0283, 117.0336	[15, 39, 43, 45]
78	16.48	Iso-angoroside C	C_36_H_48_O_19_	[M−H]^−^	783.2701	783.2717	− 2.04	783.2706, 607.2253, 193.0499, 175.0393, 160.0158	[41]
79	16.50	3-Hexenyl-*β*-D-glucopyranoside ^M^	C_12_H_22_O_6_	[M−H]^−^	261.1340	261.1344	− 1.53	262.1340, 187.0969, 125.0962	[50]
80	16.55	Astragalin	C_21_H_20_O_11_	[M−H]^−^	447.0928	447.0933	− 1.12	447.0928, 284.0324, 255.0295, 227.0345	[38]
81	16.67	isorhamnetin-3-O-glucoside	C_22_H_22_O_12_	[M + H]^+^	479.1189	479.1184	1.04	479.1189, 317.0652, 302.0414, 274.0465, 153.0179	[46]
82	16.79	Lobetyol	C_14_H_18_O_3_	[M + COOH]−	279.1236	279.1238	− 0.72	279.1236, 235.1335, 205.1227, 137.0755	[51]
83	16.84	Genistin ^M^	C_21_H_20_O_10_	[M + H]^+^	433.1122	433.1129	− 1.62	433.1122, 271.0596, 243.0651, 153.0180	[46]
84	16.96	Tangshenoside V	C_21_H_26_O_12_	[M−H]^−^	469.1334	469.1351	− 3.62	469.1334, 367.1006, 325.0924, 271.0605, 163.0392, 119.0492	[50]
85	17.00	Naringenin-6-C-glucoside	C_21_H_22_O_10_	[M−H]^−^	433.1133	433.1140	− 1.62	433.1133, 271.0601, 151.0028, 119.0493	[41]
86	17.05	6-Methoxyluteolin 3'-Glucoside	C_22_H_22_O_11_	[M−H]^−^	461.1083	461.1089	− 1.30	461.1083, 283.0245, 255.0295	[41]
87	17.22	3,5-O-Dicaffeoyl quinic acid	C_25_H_24_O_12_	[M−H]^−^	515.1189	515.1195	− 1.16	515.1189, 353.0875, 191.0554, 179.0342, 135.0442	[47]
88	17.26	Naringenin 4'-O-glucoside	C_21_H_22_O_10_	[M−H]^−^	433.1131	433.1140	− 2.08	433.1131, 271.0610, 151.0028, 119.0493	[41]
89	17.27	Scrophulide	C_30_H_38_O_15_	[M−H]^−^	637.2131	637.2138	− 1.10	637.2131, 161.0236	[42]
90	17.36	Genistein	C_15_H_10_O_5_	[M−H]^−^	269.0453	269.0455	− 0.74	269.0453,255.0603, 225.0553, 153.0184, 133.0285	[46]
91	17.37	Rhamnocitrin	C_16_H_12_O_6_	[M + H]^+^	301.0702	301.0707	− 1.66	301.0702, 245.0801, 231.06549, 167.0336	[14, 41]
92	17.49	Lobetyolin	C_20_H_28_O_8_	[M + COOH]−	441.1764	441.1766	− 0.45	441.1764, 279.0669, 215.1068, 185.096	[39]
93	17.51	Liquirtin apioside	C_26_H_30_O_13_	[M−H]^−^	549.1606	549.1614	− 1.46	549.1606, 417.1208, 255.0659, 153.0184, 119.0492	[41]
94	17.83	4,5-O-Dicaffeoyl quinic acid	C_25_H_24_O_12_	[M−H]^−^	515.1200	515.1195	0.97	515.1200, 353.0875, 191.0555, 173.0447	[47]
95	17.85	3-Butyl-4-hydroxyphthalide	C_12_H_14_O_3_	[M + H]−	207.1014	207.1016	− 0.97	207.1014, 189.0909, 161.0960	[16]
96	18.17	Naringenin 7-O-glucoside ^M^	C_21_H_22_O_10_	[M−H]^−^	433.1123	433.1140	− 3.93	433.1123, 271.0611, 151.0028, 119.0492	[41]
97	18.18	Naringenin	C_15_H_12_O_5_	[M + H]^+^	273.0753	273.0757	− 1.46	273.0753, 153.0181, 147.0439, 119.0492	[41, 44]
98	18.32	Neoisoliquiritin ^M^	C_21_H_22_O_9_	[M−H]^−^	417.1189	417.1191	− 0.48	417.1189, 255.0660, 135.0078	[18]
99	18.40	Z-harpagoside	C_24_H_30_O_11_	[M−H]^−^	493.1709	493.1715	− 1.22	493.1709, 345.1181, 183.0656, 147.0443	[41]
100	18.41	Ononin	C_22_H_22_O_9_	[M + H]^+^	431.1335	431.1337	− 0.46	431. 1335, 269. 0806, 254.0573, 213.0912, 118.0416	[14]
101	18.54	Senkyunolide I ^# M^	C_12_H_16_O_4_	[M + H]^+^	225.1119	225.1121	− 0.89	225.1119, 207.1013, 189.0906, 179.1059, 161.0958	[16]
102	18.63	Cistanoside D	C_31_H_40_O_15_	[M−H]^−^	651.2281	651.2294	− 2.00	652.2371, 193.0502, 175.0394	[42]
103	19.01	Harpagoside ^#^	C_24_H_30_O_11_	[M−H]^−^	493.1703	493.1715	− 2.43	493.1703, 345.1181, 183.0656, 147.0443	[41]
104	19.15	Senkyunolide F	C_12_H_14_O_3_	[M + H]^+^	207.1013	207.1016	− 1.45	207.1013, 189.0907, 161.0962	[16]
105	19.16	Astragaloside VII isomer	C_47_H_78_O_19_	[M + COOH]−	991.5122	991.5119	0.30	991.5122, 945.5055, 783.4508	[41]
106	19.36	Isomucronulatol 7-O-glucoside	C_23_H_28_O_10_	[M−H]^−^	463.1616	463.1610	1.30	463.1616, 301.1078, 286.0844, 179.0706,135.0442	[52]
107	19.46	Licochalcone B	C_16_H_14_O_5_	[M−H]^−^	285.0764	285.0768	− 1.40	285.0764, 270.0530, 177.0183, 150.0313	[18]
108	19.50	Emodin	C_15_H_10_O_5_	[M−H]^−^	269.0453	269.0455	− 0.74	269.0453, 241.0504, 213.0547	[40, 43]
109	19.55	Baicalin	C_21_H_18_O_11_	[M−H]^−^	445.0770	445.0776	− 1.35	445. 0770, 269. 0454, 251. 0340, 223. 0394	[46]
110	19.92	6-Methoxyluteolin 7-Glucoside	C_22_H_22_O_11_	[M−H]^−^	461.1080	461.1089	− 1.95	461.1080, 299.0558, 283.0245, 255.0295	[41]
111	20.12	Coumarin	C_9_H_6_O_2_	[M + H]^+^	147.0438	147.0441	− 2.04	147.0438, 119.0548	[46]
112	20.30	Isoliquiritigenin	C_15_H_12_O_4_	[M + H]^+^	257.0804	257.0808	− 1.56	257.0804, 239.0701, 147.0439, 137.0232, 119.0492	[18]
113	20.30	Calycosin	C_16_H_12_O_5_	[M−H]^−^	283.0609	283.0612	− 1.06	283.0609, 268.0374, 223.0397, 148.0158, 135.0081	[18, 41]
114	20.40	Luteolin ^# M^	C_15_H_10_O_6_	[M + H]^+^	287.0544	287.0550	− 2.09	287.0544, 153.0180, 135.0439	[38, 43]
115	20.46	Licoricesaponin G2 or isomer	C_42_H_62_O_17_	[M + H]^+^	839.4050	839.4060	− 1.19	839.4050, 469.3307	[14]
116	20.73	Astragaloside VII	C_47_H_78_O_19_	[M + COOH]−	991.5122	991.5119	0.30	991.5122, 945.5055, 783.4508	[41]
117	21.02	senkyunolide C ^M^	C_12_H_12_O_3_	[M + H]^+^	205.0855	205.0859	− 1.95	205.0855, 187.0752, 149.0232	[16]
118	21.24	2-Hydroxyethyl 4-methoxycinnamate	C_12_H_14_O_4_	[M−H]^−^	221.0812	221.0819	− 3.17	221.0812, 177.0913	[42]
119	21.35	Cnidilide	C_12_H_18_O_2_	[M + H]^+^	195.1378	195.1380	− 1.02	195.1378, 177.1271, 149.1322	[17]
120	21.68	Astragaloside IV ^#^	C_41_H_68_O_14_	[M + COOH]−	829.4580	829.4591	− 1.33	829.4580,783.4532,489.3586, 161.0449, 131.0234, 101.0234	[41]
121	21.71	Wogonoside	C_22_H_20_O_11_	[M + H]^+^	461.1062	461.1078	− 3.47	461.1062, 285.0753, 270.0518	[46]
122	21.90	Ginsenoside Ro ^#^	C_48_H_76_O_19_	[M−H]^−^	955.4904	955.4908	− 0.42	955.4904, 793.4373, 731.4380, 569.3840, 523.3788	[37]
123	22.23	Apigenin	C_15_H_10_O_5_	[M + H]^+^	271.0597	271.0601	− 1.48	271.0597, 243.0645, 215.0701, 153.0181	[14, 46]
124	22.28	Licoricesaponin G2	C_42_H_62_O_17_	[M + H]^+^	839.4050	839.4060	− 1.19	839.4050, 469.3308	[14]
125	22.29	Neocnidilide	C_12_H_18_O_2_	[M + H]^+^	195.1378	195.1380	− 1.02	195.1378, 177.1271, 149.1324, 135.1167	[17]
126	22.35	9,12,13-trihydroxy − 10-octadecenoic acid ^M^	C_18_H_34_O_5_	[M−H]^−^	329.2330	329.2333	− 0.91	329.2330, 229.1441, 211.1334	[39]
127	22.41	Baicalein or Norwogonin	C_15_H_10_O_5_	[M + H]^+^	271.0598	271.0601	− 1.11	271.0598, 243.0645, 215.0701, 153.0181	[46]
128	22.81	Calycosin isomer	C_16_H_12_O_5_	[M−H]^−^	283.0610	283.0612	− 0.71	283.0610, 268.0375, 224.0474, 211.0391, 135.0078	[41]
129	23.21	Astragaloside II ^#^	C_43_H_70_O_15_	[M + COOH]−	871.4697	871.4697	0.00	871.4697, 825.4590, 765.4442, 179.0560	[41]
130	23.98	Pinocembrin ^M^	C_15_H_12_O_4_	[M−H]^−^	255.0657	255.0663	− 2.35	255.0657, 135.0078, 119.0493	[41]
131	24.14	Gigantol	C_16_H_18_O_4_	[M + H]^+^	275.1275	275.1278	− 1.09	275.1275, 151.0752, 135.0596, 119.0493	[44]
132	24.36	Vestitol	C_16_H_16_O_4_	[M−H]^−^	271.0976	271.0976	0.00	271.0976, 256.0747, 149.0599, 135.0443	[18]
133	24.41	Formononetin ^M^	C_16_H_12_O_4_	[M−H]^−^	267.0660	267.0663	− 1.12	267.0660, 252.0424, 223.0395, 195.0444	[18]
134	24.67	Z-6,7-epoxyligustilide	C_12_H_14_O_3_	[M + H]^+^	207.1012	207.1016	− 1.93	207.1012, 189.0908, 171.0802, 161.0958, 133.0283	[16]
135	24.74	Licoricesaponin E2	C_42_H_60_O_16_	[M−H]^−^	819.3803	819.3809	− 0.73	819.3803, 351.0562, 193.0347	—
136	24.85	Glyasperins M	C_21_H_20_O_6_	[M + H]^+^	369.1327	369.1333	− 1.63	369.1327, 313.0702, 285.0751, 271.0598	[14]
137	24.86	Uralsaponin B	C_42_H_62_O_16_	[M−H]^−^	821.3963	821.3965	− 0.24	821.3963, 351.0569, 193.0348	[41]
138	24.90	Soyasaponin I	C_48_H_78_O_18_	[M + H]^+^	943.5245	943.5261	− 1.70	943.5245, 599.3925, 441.3721, 423.3611	[41]
139	25.08	Isomucronulatol	C_17_H_18_O_5_	[M + H]^+^	303.1221	303.1227	− 1.98	303.1221, 193.0860, 181.0860, 167.0701, 123.0441	[46]
140	25.09	Senkyunolide D ^M^	C_12_H_14_O_4_	[M−H]^−^	221.0814	221.0819	− 2.26	221.0814, 177.0913	[16]
141	25.44	Glyasperins B	C_21_H_22_O_6_	[M + H]^+^	371.1496	371.1489	1.89	371.1496, 353.1356, 315.0857, 297.0746, 175.0388, 147.0439	[18]
142	25.47	Glycyrrhizic acid ^# M^	C_42_H_62_O_16_	[M−H]^−^	821.3961	821.3965	− 0.49	821.3961, 469.3346, 351.0563, 193.0347	[41]
143	25.57	Macranthoside A	C_47_H_76_O_17_	[M−H]^−^	911.5007	911.5010	− 0.33	911.5007, 821.4020, 702.8591	[47]
144	26.57	Senkyunolide C isomer	C_12_H_12_O_3_	[M + H]^+^	205.0858	205.0859	− 0.49	205.0858, 187.0752, 149.0233	[16]
145	26.71	Licorice saponine K2	C_42_H_62_O_16_	[M−H]^−^	821.3958	821.3965	− 0.85	821.3958, 351.0570, 193.0348	[41]
146	26.81	Glycycoumarin	C_21_H_20_O_6_	[M + H]^+^	369.1328	369.1333	− 1.35	369.1328, 313.0703, 285.0754	[14]
147	26.90	Isoastragaloside I	C_45_H_72_O_16_	[M + COOH]−	913.4757	913.4802	− 4.93	913.4757, 867.4725, 513.1759	[41]
148	27.29	Glyasperins C	C_21_H_24_O_5_	[M−H]^−^	355.1559	355.1551	2.25	355.1559, 323.1285, 229.0866, 125.0234	[18]
149	27.45	Butylphthalide ^M^	C_12_H_14_O_2_	[M + H]^+^	191.1064	191.1067	− 1.57	191.1064, 173.0959, 145.1011, 117.0700	[16]
150	27.47	Senkyunolide E	C_12_H_12_O_3_	[M−H]^−^	203.0707	203.0714	− 3.45	203.0707, 174.0314 130.0414	[16]
151	27.87	Chikusetsu saponin IVa	C_42_H_66_O_14_	[M−H]^−^	793.4370	793.4380	− 1.26	793.4370, 613.3704, 569.3859, 455.3530	[37]
152	28.20	Atractylenolide III ^#^	C_15_H_20_O_3_	[M + H]^+^	249.1477	249.1485	− 3.21	249.1477,231.1378, 175.0754, 163.0752	[39]
153	28.61	Senkyunolide A	C_12_H_16_O_2_	[M + H]^+^	193.1222	193.1223	− 0.52	193.1222, 175.1116, 147.1168, 137.0596, 119.0856	[16]
154	28.63	Licoricone	C_22_H_22_O_6_	[M + H]^+^	383.1475	383.1489	− 3.65	383.1475, 327.0858, 299.0911	[18]
155	28.64	Isolicoflavonol	C_20_H_18_O_6_	[M−H]^−^	353.1027	353.1031	− 1.13	353.1027, 227.0709, 125.0235	[18]
156	29.19	Glycyrol	C_21_H_18_O_6_	[M−H]^−^	365.1027	365.1031	− 1.10	365.1027, 307.0247, 295.0245	[18]
157	29.47	*α*-Linolenic acid ^M^	C_18_H_30_O_2_	[M + H]^+^	279.2314	279.2319	− 1.79	279.2314, 261.2206, 149.0232	[16]
158	30.81	E-ligustilide	C_12_H_14_O_2_	[M + H]^+^	191.1062	191.1067	− 2.62	191.1065, 173.0961, 145.1012	[16]
159	31.11	Glycyrin	C_22_H_22_O_6_	[M + H]^+^	383.1483	383.1489	− 1.57	383.1483, 327.0858, 299.0910	[18]
160	31.23	Z-ligustilide	C_12_H_14_O_2_	[M + H]^+^	191.1065	191.1067	− 1.05	191.1065, 173.0959, 145.1011, 117.0700	[16]
161	32.18	Atractylenolide II ^# M^	C_15_H_20_O_2_	[M + H]^+^	233.1532	233.1536	− 1.72	233.1532, 215.1425, 187.1479, 151.0753, 105.0700	[39]
162	32.41	Glyasperins D	C_22_H_26_O_5_	[M + H]^+^	371.1836	371.1853	− 4.58	371.1836, 315.1221, 303.1221,235.1327, 167.0701, 123.0441	[18]
163	35.67	18-*β*-Glycyrrhetinic acid ^M^	C_30_H_46_O_4_	[M + H]^+^	471.3465	471.3469	− 0.85	471.3465, 453.3361, 425.3408, 317.2114	[14]
164	36.43	Levistilide A	C_24_H_28_O_4_	[M + H]^+^	381.2056	381.2060	− 1.05	381.2056, 191.1065	[16]

^#^Confirmed with reference standards; ^M^indicated components into the serum

**Fig. 3:**
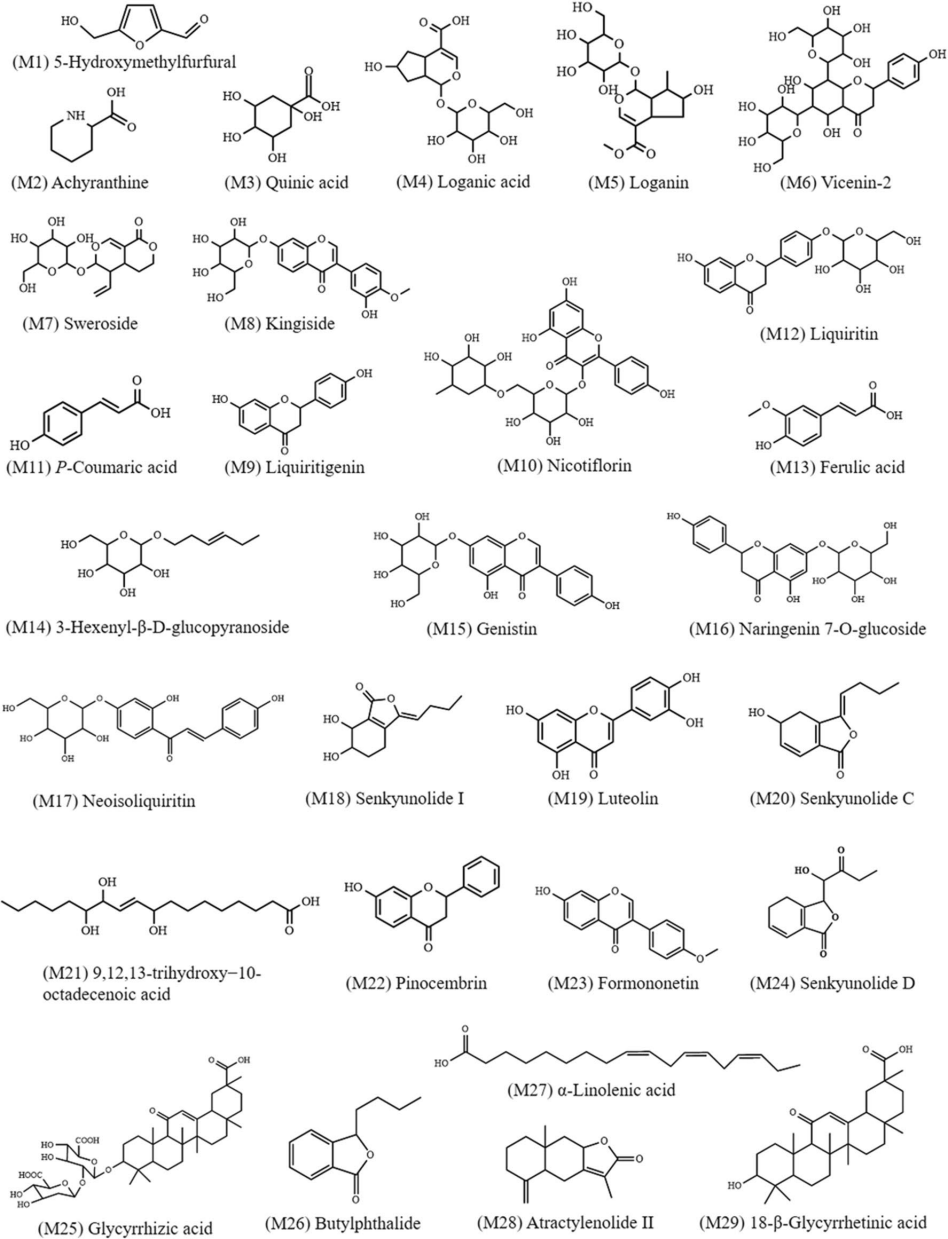
Chemical structures of the 29 prototype compounds absorbed in TSMT-derived serum

**Fig. 4:**
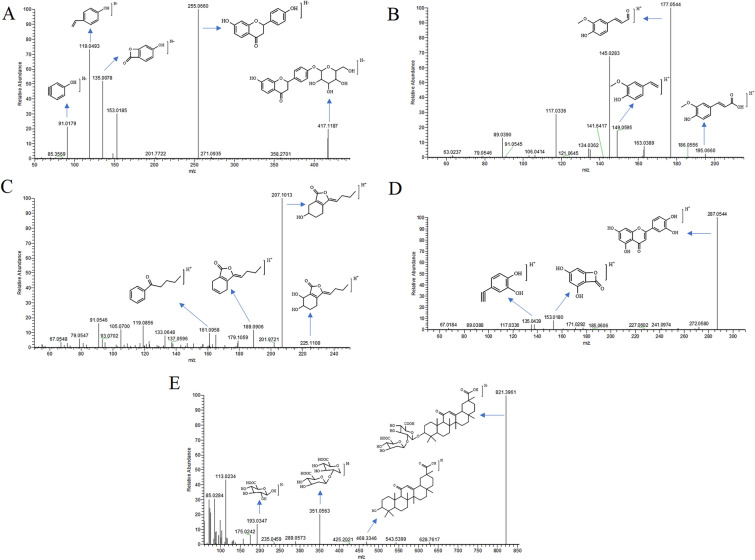
Proposed mass fragments and fragmentation pathways of Liquiritin (**A**), Ferulic acid (**B**), Senkyunolide I (**C**), Luteolin (**D**), and Glycyrrhizic acid (**E**)

A total of 29 constituents were generated from TSMT and absorbed into the serum. These were identified as prototype substances and their chemical structures were presented in Fig. 3. The selected crucial and representative fragmentation pathways of seven compounds absorbed into serum were analyzed in Fig. 4. For example, compound 68 (M12) was identified as Liquiritin at the retention time of 15.63 min, with the excimer ion peak of m/z 417.1187 [M–H]^−^ (C_21_H_22_O_9_) by compared with RS and related study [14]. The base peak was formed at m/z 255.0660 through the loss of a 162 Da glucose residue (–C_6_H_10_O_5_) from the protonated ion. Also, another major fragment ion was produced at m/z 135.0078 (C_7_H_3_O_3_) by simultaneous loss of 120 Da (–C_8_H_8_O) from m/z 255.0660. Further, the product ions were also obtained at m/z 119.0493 (–C_8_H_7_O) and m/z 91.0179. The cracking pattern of Liquiritin belonging to GC was shown in Fig. 4A. Similarly, compound 77 (M13) was characterized as Ferulic acid at the retention time of 16.39 min and the excimer ion peak at m/z 195.0660 [M + H]^+^ (C_10_H_10_O_4_) by compared with RS and many other studies [15]. The product ion was produced at m/z 177.0544 via elimination of a residue of one water molecule (18 Da). Another major fragment ion was produced at m/z 149.0595 by successive removal of one CO molecule (28 Da) from m/z 177.0541. The detailed cleavage information of ferulic acid derived from DG was displayed in Fig. 4B. Compound 101 (M18) was unequivocally identified as Senkyunolide I with 18.54 min by comparison with an authentic standard and a previous study [16], which produced a dominant deprotonated ion [M + H]^+^ at m/z 225.1119 (C_12_H_16_O_4_). A product ion was also observed at m/z 207.1013 and m/z 189.0906 presumably resulting from the loss of one and two H_2_O molecule, respectively. Furthermore, the base peak was formed at m/z 161.0958 by the loss of two water and one CO molecules from the protonated ion. The information on fragment ions information of Senkyunolide I originating from DG was characterized in Fig. 4C. Compound 114 (M19) exhibited an [M + H]^+^ ion at m/z 287.0544 with the retention time of 20.40 min, which corresponded to a unique elemental composition of C_15_H_10_O_6_. Comparing our data with the previous report, the compound was identified as Luteolin [17]. The product ion at m/z 153.0180 and m/z 135.0439 was respectively obtained by the loss of 134 Da (–C_8_H_6_O_2_), and 152 Da (–C_7_H_4_O_4_) chemical structures from the protonated molecular ion of m/z 287.0544, respectively. The above-mentioned product ion of Luteolin derived from JYH was displayed in Fig. 4D. Compound 142 (M25) was consistent with the main characteristics of Glycyrrhizic acid (C_42_H_62_O_16_), as per the comparison with RS and the related literature [14, 18]. It displayed an [M–H]^−^ ion at m/s 821.3961 with a retention time of 25.47 min. In the subsequent MS cleavage process, a chemical structure of 352 Da (–C_12_H_16_O_12_) was removed from the quasi-molecular ion to form the characteristic fragment ion at m/z 469.3346, which corresponded to a molecular structure of Glycyrrhetinic acid. Simultaneously, the product ion was also detected at m/z 193.0347 through the loss of a chemical structure of 158 Da (–C_6_H_6_O_5_) from the ion at m/z 351.0563. The proposed fragmentation of Glycyrrhizic acid derived from GC was given in Fig. 4E.

### Network pharmacology analysis

#### Collection of key therapeutic targets and analysis of PPI construction

**Fig. 5:**
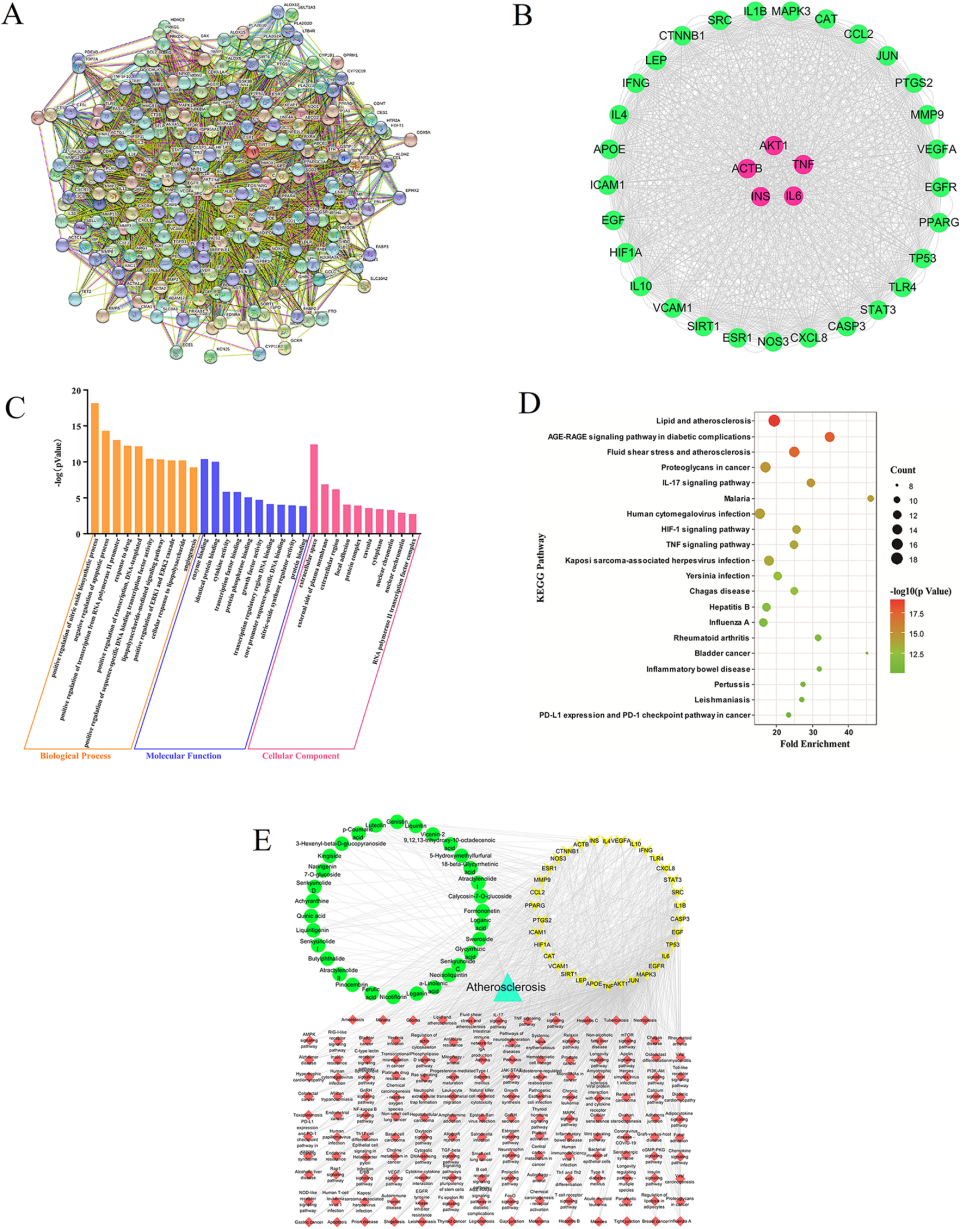
Network pharmacology analysis of 29 serum-absorbed constituents of TSMT showing PPI network of 236 disease-drug targets (**A**), the module of selection of 34 hub targets (**B**), GO analysis (**C**), KEGG pathway enrichment (**D**) and the “Compounds-Targets -Pathways-Disease” network of TSMT in anti-AS mechanism (**E**) (This network consisted of 215 nodes and 1170 edges. the green elliptical nodes represented 29 active therapeutic ingredients, yellow V Triangle nodes represented 34 overlapped hub gene, red diamond nodes represented 151 signaling pathways and the bright green triangle node represented atherosclerosis disease)

The absorbed compounds were used to predict the potential anti-AS targets and pathways of TSMT via our network pharmacology tool, whose results are shown in Table 1. The compounds or disease targets were searched in the databases and 236 intersection targets were screened out. These targets were imported into the STRING database and the relationship of Protein–Protein-Interaction (PPI) targets was documented in Fig. 5A, initially. This data was further imported into Cytoscape3.7.1 software for Network Analyze with three important topological parameters of Degree, Betweenness, and Closeness. Among them, Degree is considered of great importance as it was the center of the node and reflected connectedness of targets participating in the pathological process of diseases, implying that a larger degree value indicated more involvement in the disease process. Ultimately, 34 targets with higher protein interaction relationships (degree value greater than 99) we visualized and selected. Moreover, the top 3 targets namely the IL-6 (Degree = 182), TNF (Degree = 182), and AKT1 (Degree = 180), showed the greatest correlation with the active compounds useful in the treatment of AS. The detailed network topology of the 34 therapeutic targets was shown in Fig. 5B and Additional file 1: Table S1. Therefore, these 34 therapeutic targets were considered as the core targets of TSMT in the treatment of AS.

#### Enrichment analysis of GO gene function and KEGG pathway

To clarify the underlying anti-AS mechanism associated with TSMT therapeutic compounds, GO enrichment and KEGG pathway analyses were performed in combination with overlapped gene analysis. The GO gene analysis (detailed information given in Additional file 1: Table S2) suggested that 34 key target functions were related to biological process, molecular function and cellular components, such as enzyme binding, identical protein, etc. While, the KEGG enrichment analysis indicated that TSMT may be involved in 151 potential pathways (*p* value < 0.01), including the five predominant pathways involving lipid and atherosclerosis, fluid shear stress and atherosclerosis, IL-17 signaling pathway, HIF-1 signaling pathway, and TNF signaling pathway (detailed information given in Additional file 1: Table S3). Interestingly, R 4.0.3 software was used to visualize the top 10 GO categories and 20 KEGG pathways, which were presented in Fig. 5C and D, respectively. The enrichment analysis revealed that 29 compounds validated in the TSMT serum samples may play crucial roles in the treatment of AS by adjusting the above-mentioned diversified biological processes and signaling pathways.

### Selection of the quality control (QC) markers

To explore the QC markers, a “components-key targets-functional pathways-disease” network was constructed using Cytoscape 3.7.1 software, which is represented in Fig. 5E. The Network Analyze was used to analyze the network relationship, which is displayed in Additional file 1: Table S4. With the degree value of greater than or equal to four, 18 potentially effective constituents were preliminarily screened out, which included Ferulic acid, Genistin, Glycyrrhizic acid, 18-*β*-Glycyrrhetinic acid, Luteolin, Formononetin, *α*-Linolenic acid, Liquiritigenin, *ρ*-Coumaric acid, Senkyunolide C, Pinocembrin, Liquiritin, Neoisoliquiritin, Atractylenolide II, Quinic acid, Loganin, 3-Hexenyl-beta-D-glucopyranoside, and Senkyunolide I. For example, Ferulic acid with 15 nodes showed the maximum degree of correlation followed by the Glycyrrhizic acid and Genistin showing the same relation degree at 12 nodes.

According to the "specificity", "efficacy correlation of TCM theory" and "measurability", of substances [19], Ferulic acid is tested as the quality standard marker for DG while Luteolin was the glycoside of cynaroside which is the quality marker of JYH. Liquiritin and Glycyrrhizic acid ate both derived from the quality standard marker of DC and are specified in the *Chinese Pharmacopoeia* (2020 edition). Additionally, DG is a sovereign drug imparting better benefits to the quality evaluation of TSMT. It consists of 5 potentially selected effective constituents, namely Senkyunolide I, Senkyunolide C, Senkyunolide D, Butylphthalide and *α*-Linolenic acid. Although, Senkyunolide I in TSMT could be measured by the current HPLC method, and Senkyunolide C, Senkyunolide D, Butylphthalide and *α*-Linolenic acid could not be detected possibly due to the polarity and low content of these chemical components in TSMT. Hence, to screen potential QC markers using network pharmacology, we selected 5 out of 29 common potentially effective anti-AS ingredients, including Liquiritin, Ferulic acid, Senkyunolide I, Luteolin, and Glycyrrhizic acid. Based on the degree of network, professional knowledge and literature validation, these five ingredients were considered potential quality markers of TSMT.

### Simultaneous quantitative analysis of the five potentially effective ingredients

**Fig. 6:**
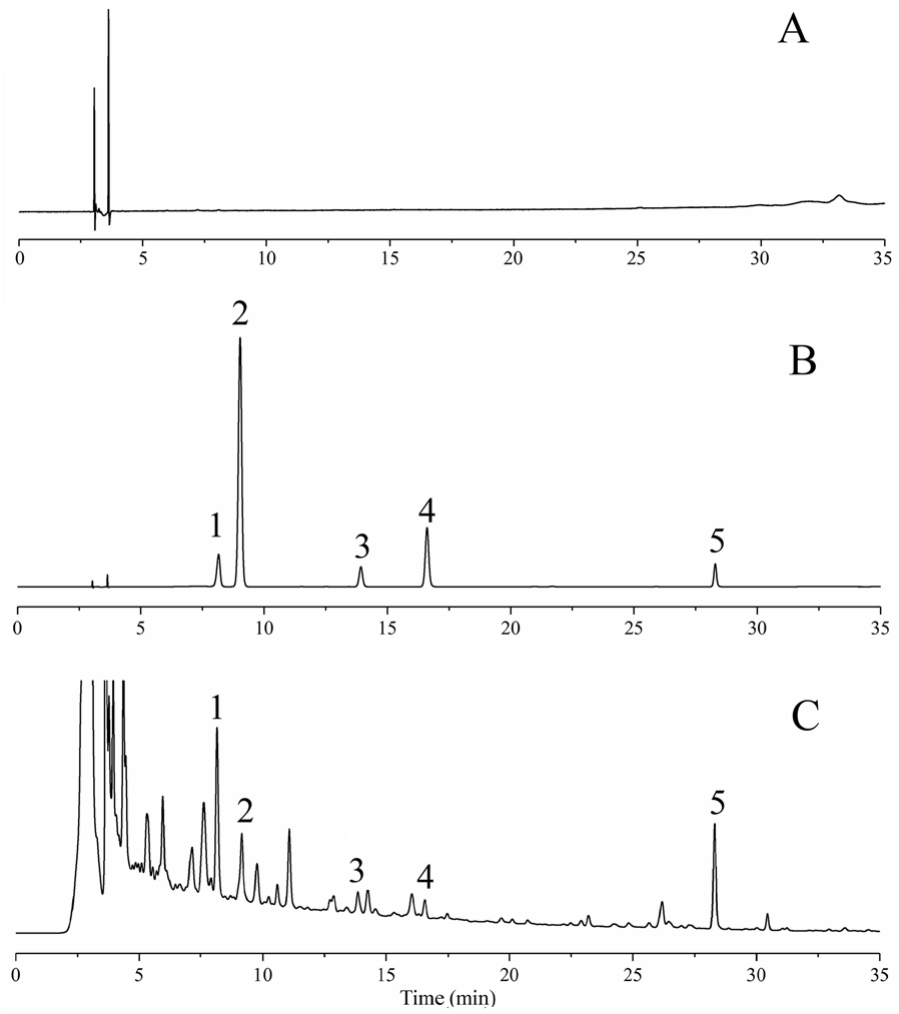
HPLC chromatograms of negative control (**A**), mixed reference substance (**B**), and TSMT samples (**C**). Peak 1–5 represented Liquiritin, Ferulic acid, Senkyunolide I, Luteolin, and Glycyrrhizic acid, respectively

To select effective components through network pharmacological prediction, we considered multifaceted factors including the detection limit of the HPLC instrument, content levels in the TSMT components, and the measurability and availability of components. Next, we chose five active constituents including Liquiritin, Ferulic acid, Senkyunolide I, Luteolin, and Glycyrrhizic acid to establish the quality standards of multiple batches of TSMT by simultaneously determining their contents. Meanwhile, the obtained chromatographic peaks of these five components showed good resolution and detection performance due to the optimized HPLC conditions. Since the calibration curves of all reference substances were linear in the determinable range (r > 0.9997), the validation of the method was verified. Additionally, this measurable method also showed satisfactory precision, repeatability, and stability of all relative analytes because the relative standard deviation (RSD%) of the peak area was less than 3% while the average recoveries ranged from 98.9% to 99.7%. This indicated that the quantitative determination method was feasible and reliable for simultaneous determination of the five potential active ingredient markers, which was demonstrated in Additional file 1: Table S5. The HPLC chromatogram of samples was displayed in Fig. 6.

Simultaneously, the above-described method was also applied to quantify five ingredients of TSMT. The average contents of the five components in nine batches of TSMT were showed in Table 2. The results showed that TSMT constituted Liquiritin (1.8006 ~ 1.8720 mg/g), Ferulic acid (0.6594 ~ 0.6916 mg/g), Senkyunolide I (0.7444 ~ 0.8054 mg/g), Luteolin (0.1882 ~ 0.1979 mg/g) and Glycyrrhizic acid (5.6651 ~ 5.9022 mg/g) in high quantities. Generally, 70% of the mean value is considered as the standard limit for the component content of Chinese patent medicines. Thereby, TSMT may contain Liquiritin, Ferulic acid, Senkyunolide I, Luteolin, and Glycyrrhizic acid in quantity not less than 1.2798, 0.4716, 0.5419, 0.1349, 4.0386 mg/g, respectively, indicating that these five compounds may serve as the major bioactive markers of TMST.

**Table 2:** Content determination of the five markers in nine batches of TSMT

No.	Liquiritin (mg/g)	Ferulic acid (mg/g)	Senkyunolide I (mg/g)	Luteolin (mg/g)	Glycyrrhizic acid (mg/g)
S1	1.8195	0.6594	0.7801	0.1910	5.7449
S2	1.8148	0.6722	0.7738	0.1921	5.7317
S3	1.8102	0.6916	0.7797	0.1928	5.7632
S4	1.8195	0.6796	0.7444	0.1882	5.7428
S5	1.8338	0.6746	0.7782	0.1939	5.7763
S6	1.8500	0.6868	0.8054	0.1983	5.8477
S7	1.8720	0.6618	0.7634	0.1979	5.9022
S8	1.8341	0.6718	0.7807	0.1920	5.7520
S9	1.8006	0.6660	0.7621	0.1895	5.6651

### Establishment of chemical fingerprints in nine batches of TSMT

**Fig. 7:**
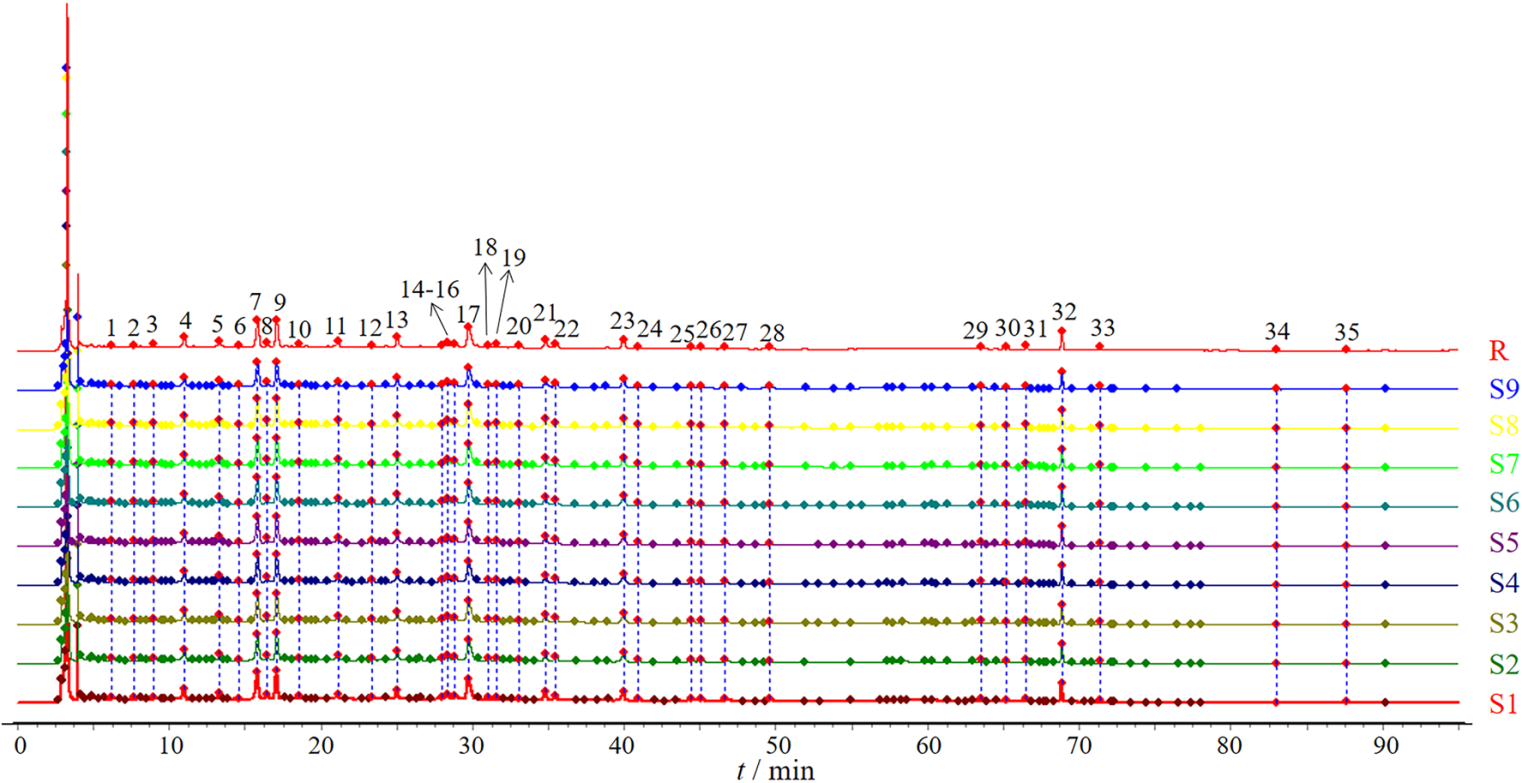
HPLC fingerprints of the reference (R) and S1–S9 in TSMT samples (R indicates fingerprints of reference; S1–S9 represents nine batches of TSMT sample in sequence)

The optimized chromatographic conditions resulted in the good separation of the constituents in all samples. Also, the results of methodology validation were satisfactory with high precision, good repeatability and high stability. Since the RSD% values of precision, repeatability, and stability in the S1 sample were less than 3%, it was beneficial for the fingerprint analysis of TSMT. Fingerprint analysis was established based on HPLC data of nine batches of TSMT (S1–S9). All the data were entered into the Similarity Evaluation System for Chromatographic Fingerprint of Traditional Chinese Medicines (2012 version) to obtain common peaks with a peak area of more than 0.09% of the total peak area during the match. A total of 35 common peaks (peak 1–35) were found in these chromatograms along with a reference fingerprint (R) spectrum (Fig. 7).

The similarity values of HPLC fingerprints were matched by calculating correlative coefficients between the TSMT samples and reference fingerprint (R). The similarities were evaluated at a value greater than 0.998 (detailed similarity analysis displayed in Additional file 1: Table S6). Based on the known reference substances and their medicinal origin, common peaks of 14, 17, 23, 28, and 32 were identified as Ferulic acid (belonging to DG), Liquiritin (belonging to GC), Senkyunolide I (belonging to DG), Luteolin (belonging to JYH), and Glycyrrhizic acid (belonging to GC), respectively. Therefore, a fingerprint analysis with a good profile of chromatographic peaks indicated a stable prescription process while the main chemical constituents of the different batches of TSMT prescription showed no significant influence overall.

## Discussion

According to the TCM theory, Tongsaimai tablet (TSMT), a commercial Chinese patent medicine, exhibited a definite medicinal effect of promoting blood circulation, dredging collaterals, tonifying qi, and nourishing Yin. The current quality standards of TSMT by CFDA (YBZ08372004) suggest chlorogenic acid, derived from JYH, as the content determination index [10]. However, the quantitative determination of only chlorogenic acid cannot fully reflect the TSMT quality. Also, the QC markers of most TCM prescriptions are selected based on the specificity of compounds mentioned in Chinese Pharmacopoeia (2020 edition). The quality standard for DG was established by determining the content of Ferulic acid in TSMT using HPLC. The quality control index compound of JYH is Cynaroside which produces an aglycon of Luteolin. Similarly, Harpagoside and Harpagoside, Astragaloside IV and Calycosin-7-glucoside, *β*-Ecdysterone, Dendrobium and Erianin, and Liquiritin and Glycyrrhizic acid can be used as marker compounds for XS, HQ, NX, SH and GC, respectively.

**Fig. 8:**
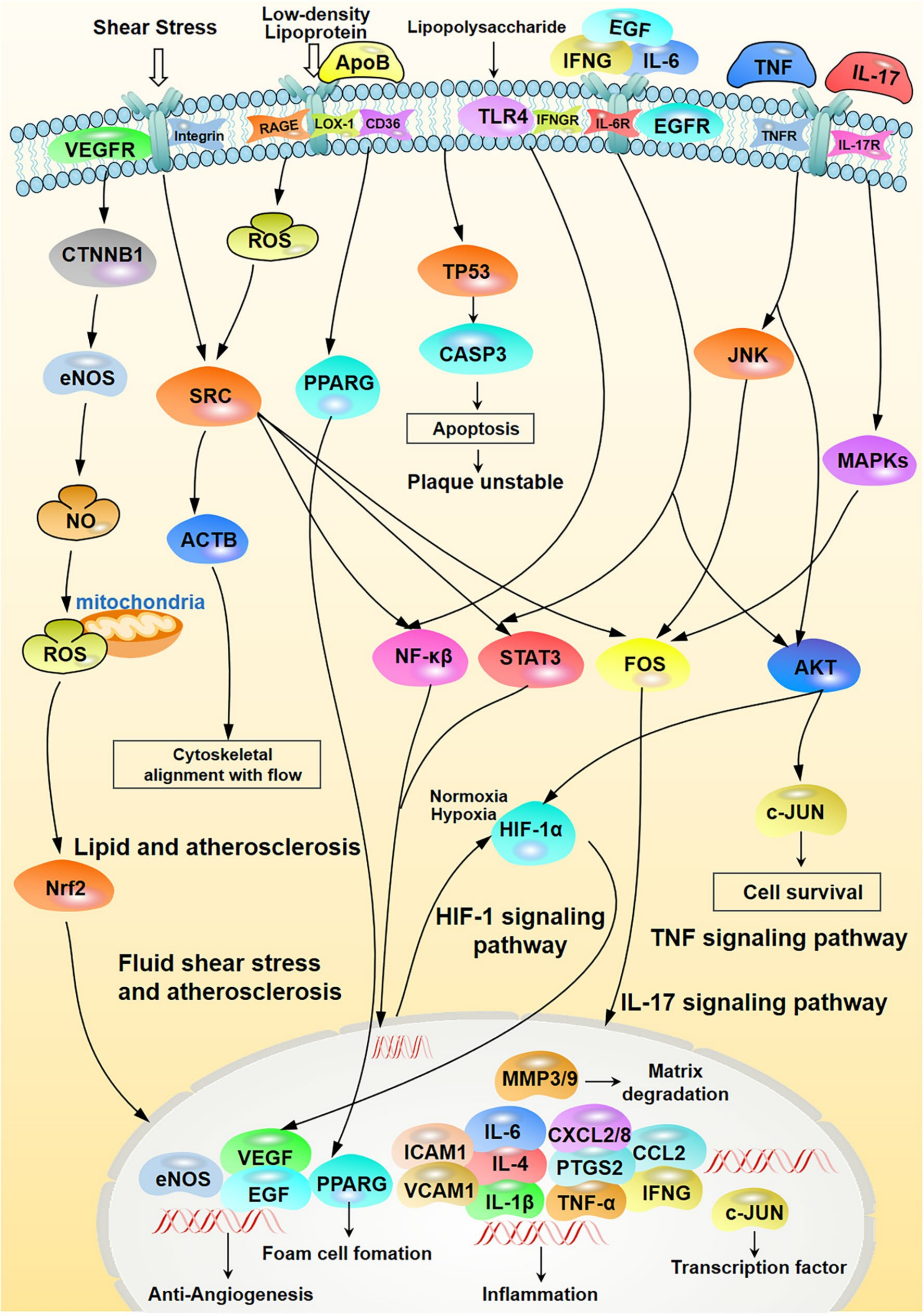
Predicted and related anti-AS mechanisms of TSMT

Atherosclerosis (AS) is a chronic vascular disease whose pathogenic mechanism begins with endothelial cell (EC) damage occurring due to lipid accumulation in blood vessels, followed by abnormal lipid metabolism of cholesterol including low-density lipoprotein (LDL) and recruitment of macrophages to the arterial walls [20]. The deposition of subendothelial lipoproteins leads to the aggregation of monocytes, that later differentiate into macrophages in the intimal compartment [21]. Macrophages uptakes oxidation-modified lipoproteins such as oxidized low-density lipoproteins (ox-LDLs) excessively and disorderly which mediated by scavenger receptors to form cholesterol-overloaded foam cells [22]. Furthermore, these foam cells trigger a series of inflammatory reactions, contributing to the necrotic core formation of atheromatous plaques. In this study, we identified 29 key active ingredients related with 34 core genes and 151 signaling pathways. Next, we constructed the pharmacological network of the anti-AS mechanism of TSMT. Consequently, we found that the function of AS was improved by five central pathways, including lipid and atherosclerosis, fluid shear stress and atherosclerosis, IL-17 signaling pathway, HIF-1 signaling pathway, and TNF signaling pathway, which were found closely related to the different regulations of anti-angiogenesis, foam cell formation, cytoskeletal alignment with the flow, inflammation, matrix degradation, and plaque instability by apoptosis (Fig. 8). Our analysis further clarified the potential mechanisms of TSMT against AS and demonstrated that the chief association of these anti-AS pathways were chiefly associated with the main response of lipid regulation, fluid shear stress, and inflammation. Lipid and atherosclerosis pathway is found significantly associated with lipid metabolism. Consistent with various studies [7–9], our results indicated that TSMT may improve the lipid disorder effect by inhibiting lipid plaque through close association with anti-inflammatory activities and antioxidative stress. Shear stress (also called frictional force), a local hemodynamic factor, is generated on the endothelial cell surface by blood flow. Low shear stress (LSS) is prevalent at the AS-prone sites [23]. Some studies have emphasized that focus on the early stages of AS, where anti-inflammatory and anti-oxidative activities could together regulate LSS [24, 25]. Moreover, IL-17 signaling pathways and TNF signaling pathways also play an indispensable role in inflammatory effects. In a previous study, the activation of IL-17 signaling pathways and TNF signaling pathways regulated pro-inflammatory and pro-atherogenic cytokines such as IL-1*β*, IL-6, TNF, along with activating transcription factors such as FOS and JUN [26]. Hypoxia has been demonstrated in AS progression while HIF-1 has been found to promote intraplaque angiogenesis and the development of foam cells. HIF-1*α* modulation can stimulate multiple factors related to the secretion of Akt, VEGF, and CXCL-8, which blocks atherosclerotic plaque development and reduces intrinsic inflammatory responses [27]. Increasing evidences have demonstrated a probable association of HIF-1 signaling pathway with the formation of foam cell, inflammatory action of macrophages, and angiogenesis preventing the development of AS [28, 29]. Therefore, through the above-mentioned mechanisms, TSMT could prevent AS by regulating lipid metabolism, inflammatory response and oxidative stress, hemodynamic shear stress, anti-angiogenic effects, etc.

In our study, we initially selected five effective prototype constituents such as Ferulic acid, Liquiritin, Senkyunolide I, Luteolin, and Glycyrrhizic acid as indicators, which were obtained from the component-target network through qualitative and quantitative HPLC analysis. Modern pharmacological studies have reported that these five compounds exerted anti-AS effects. Ferulic acid has exhibited anti-atherogenic properties by significantly regulating lipid metabolism in high-fat diet-induced ApoE−/− mouse model. It also effectively improves the expression of mitochondrial function while reducing the oxidative stress during vascular damage of endothelial cells and human mononuclear cells in the mouse model of AS [30, 31]. Senkyunolide I may alleviate thrombosis as a key pathological event in cardiovascular disease, affecting the inhibition of platelet activation, coagulation cascade, oxidative stress, endoplasmic reticulum stress, and apoptosis [32, 33]. Luteolin inhibits the adhesion of TNF-*α* induced monocytes to endothelial cells by blocking CCL2, ICAM-1, and VCAM-1 expression along with suppressing the IκB*α*/NF-κB mediated signaling pathway [34]. Liquiritin performs anti-inflammatory activities in LPS-stimulated mouse brain microglia cell by inhibiting pro-inflammatory mediators such as iNOS, PTGS2, TNF-*α*, IL-1*β*, and IL-6 [35]. Glycyrrhizic acid has exerted an atheroprotective effect in AS treatment by improving lipid metabolism. It decreases the serum lipid levels and atherosclerotic plaque deposition, and further suppresses vascular inflammation in Th17 cells in ApoE−/− mice by reducing IL-6 and STAT3 phosphorylation [36].

## Conclusion

In this study, serum pharmacochemistry and network pharmacology were integrated to establish the quality assessment of TSMT along with exploring the related underlying mechanisms of TSMT in AS alleviation. The component-target-pathway network was used to screen five potentially bioactive ingredients in TSMT, which included Ferulic acid, Liquiritin, Senkyunolide I, Luteolin and Glycyrrhizic acid. These sere considered as the potential QC markers. Based on these selected indexes, HPLC–DAD analysis and HPLC fingerprint method were performed for simultaneous multi-component determination, which helped in assessing the quality of nine batches of TSMT samples. In summary, this study may provide substantial evidence to improve the quality control of TSMT, greatly facilitating the efficacy and safety of TSMT in clinical applications. To the best of our knowledge, this is the first attempt to explore the potential anti-AS mechanism and establish a quality control system to provide comprehensive quality of TSMT.

## Supplementary Information

**Additional file 1: Table S1**. Network topological analysis of 34 core therapeutic targets in PPI network. **Table S2**. TSMT improves TOP 10 GO categories of core targets of atherosclerosis. **Table S3**. TSMT alleviates TOP 20 KEGG pathway of core targets of atherosclerosis. **Table S4**. Crucial anti-AS ingredients were selected as candidate active compounds in TSMT. **Table S5**. Methodological investigation results of the content determination method of TSMT. **Table S6**. Similarity evaluation results of HPLC fingerprint of nine batches of S1-S9 in TSMT.

## Data Availability

The datasets used and/or analyzed during the current study are available from the corresponding author on reasonable request.

## Abbreviations

TSMT: Tongsaimai tablet
CFDA: China Food and Drug Administration
TCM: Traditional Chinese Medicine
AS: Atherosclerosis
QC: Quality control
HPLC: High performance liquid chromatography
DAD: Diode array detector
RSD: Relative standard deviation
