# Supplementary material for: Quality assessment and Q-markers discovery of Tongsaimai tablet by integrating serum pharmacochemistry and network pharmacology for anti-atherosclerosis benefit
Source: Chin Med. 2022 Sep 2;17:103. doi: 10.1186/s13020-022-00658-9 (PMC9438231; doi:10.1186/s13020-022-00658-9)
Supplement: Supplementary file 1 — Additional file 1: Table S1. Network topological analysis of 34 core therapeutic targets in PPI network. Table S2. TSMT improves TOP 10 GO categories of core targets of atherosclerosis. Table S3. TSMT alleviates TOP 20 KEGG pathway of core targets of atherosclerosis. Table S4. Crucial anti-AS ingredients were selected as candidate active compounds in TSMT. Table S5. Methodological investigation results of the content determination method of TSMT. Table S6. Similarity evaluation results of HPLC fingerprint of nine batches of S1-S9 in TSMT. [file 13020_2022_658_MOESM1_ESM.docx]

***Additional materials***

**Quality assessment and Q-markers Discovery of Tongsaimai tablet by integrating serum pharmacochemistry and network pharmacology for anti-atherosclerosis benefit**

Yanfen Cheng^a, †^, Meng Xiao ^a, †^, Jiamei Chen^a^, Di Wang^a^, Yichen Hu^b^, Chenfeng Zhang^c,d^, Tuanjie Wang^c,d^, Chaomei Fu^a^, Yihan Wu^a^,*, Jinming Zhang^a,^*

^a^ *State Key Laboratory of Southwestern Chinese Medicine Resources, School of Pharmacy, Chengdu University of Traditional Chinese Medicine, Chengdu, 611137, China*

^b^ *Key Laboratory of Coarse Cereal Processing, Ministry of Agriculture and Rural Affairs, Chengdu University, Chengdu 610106, Sichuan, China*

^c^ *Jiangsu Kanion Pharmaceutical CO. LTD, Lianyungang, 222001, China*

^d^ *State Key Laboratory of New-tech for Chinese Medicine Pharmaceutical Process, Lianyungang, 222001, China*

^†^ Yanfen Cheng and Meng Xiao contributed equally to this work

* **Corresponding author:**

Yihan Wu, E-mail: yihanwuone@126.com

Jinming Zhang, E-mail: cdutcmzjm@126.com

**Table S1**. Network topological analysis of 34 core therapeutic targets in PPI network

| NO. | Name | Protein names | Degree | Betweenness Centrality | Closeness Centrality |
| --- | --- | --- | --- | --- | --- |
| 1 | IL6 | Interleukin-6 | 182 | 0.036065 | 0.815972 |
| 2 | TNF | Tumor necrosis factor | 182 | 0.036689 | 0.815972 |
| 3 | AKT1 | AKT serine/threonine kinase 1 | 180 | 0.034423 | 0.810345 |
| 4 | ACTB | Beta-actin | 171 | 0.032391 | 0.785953 |
| 5 | INS | Insulin | 169 | 0.042512 | 0.780731 |
| 6 | IL1B | Interleukin-1 beta | 162 | 0.020946 | 0.762987 |
| 7 | VEGFA | Vascular endothelial growth factor A | 157 | 0.015109 | 0.750799 |
| 8 | TP53 | Cellular tumor antigen p53 | 146 | 0.02046 | 0.723077 |
| 9 | JUN | Transcription factor AP-1 | 138 | 0.014073 | 0.707831 |
| 10 | CASP3 | Caspase-3 | 137 | 0.010172 | 0.705706 |
| 11 | PTGS2 | Prostaglandin G/H synthase 2 | 136 | 0.018113 | 0.703593 |
| 12 | MAPK3 | MAP kinase-activated protein kinase 3 | 134 | 0.012352 | 0.699405 |
| 13 | STAT3 | Signal transducer and activator of transcription 3 | 134 | 0.010718 | 0.699405 |
| 14 | EGFR | Epidermal growth factor receptor | 132 | 0.014831 | 0.693215 |
| 15 | CCL2 | C-C motif chemokine 2 | 131 | 0.008885 | 0.693215 |
| 16 | MMP9 | Matrix metalloproteinase-9 | 131 | 0.00968 | 0.693215 |
| 17 | PPARG | Peroxisome proliferator-activated receptor gamma | 129 | 0.014664 | 0.68915 |
| 18 | TLR4 | Toll-like receptor 4 | 129 | 0.008876 | 0.68915 |
| 19 | CXCL8 | Interleukin-8 | 129 | 0.00815 | 0.68915 |
| 20 | SRC | Proto-oncogene tyrosine-protein kinase Src | 127 | 0.011487 | 0.685131 |
| 21 | IL10 | Interleukin-10 | 127 | 0.007468 | 0.685131 |
| 22 | HIF1A | Hypoxia-inducible factor 1-alpha | 124 | 0.00729 | 0.679191 |
| 23 | CTNNB1 | Catenin beta-1 | 122 | 0.008318 | 0.675287 |
| 24 | EGF | Epidermal growth factor | 118 | 0.006113 | 0.667614 |
| 25 | ICAM1 | Intercellular adhesion molecule 1 | 112 | 0.004708 | 0.656425 |
| 26 | IL4 | Interleukin-4 | 104 | 0.004036 | 0.638587 |
| 27 | ESR1 | Estrogen receptor 1 | 103 | 0.008166 | 0.636856 |
| 28 | CAT | Catalase | 103 | 0.009025 | 0.640327 |
| 29 | LEP | Leptin | 103 | 0.008742 | 0.640327 |
| 30 | NOS3 | Nitric oxide synthase 3 | 103 | 0.010386 | 0.640327 |
| 31 | APOE | Apolipoprotein E | 101 | 0.010992 | 0.636856 |
| 32 | IFNG | Interferon gamma | 101 | 0.002582 | 0.633423 |
| 33 | VCAM1 | Vascular cell adhesion protein 1 | 101 | 0.003765 | 0.635135 |
| 34 | SIRT1 | NAD-dependent protein deacetylase sirtuin-1 | 99 | 0.00769 | 0.633423 |

**Table S2**. TSMT improves TOP 10 GO categories of core targets of atherosclerosis

| NO. | Category | Term ID | Name | p Value | Count | Genes |
| --- | --- | --- | --- | --- | --- | --- |
| 1 | Biological Process | GO:0045429 | positive regulation of nitric oxide biosynthetic process | 6.84E-19 | 11 | IL6, IFNG, IL1B, AKT1, PTGS2, ESR1, TNF, TLR4, EGFR, ICAM1, INS |
| 2 |  | GO:0043066 | negative regulation of apoptotic process | 4.85E-15 | 16 | IL10, SRC, ANXA5, STAT3, SIRT1, MMP9, EGFR, VEGFA, IL4, IL6, CASP3, LEP, CAT, ALB, AKT1, TP53 |
| 3 |  | GO:0045944 | positive regulation of transcription from RNA polymerase II promoter | 9.51E-14 | 19 | IL10, JUN, STAT3, HIF1A, SIRT1, ESR1, TNF, EGFR, VEGFA, IL4, IL6, IFNG, IL1B, AKT1, CTNNB1, PPARG, TP53, TLR4, MAPK3 |
| 4 |  | GO:0042493 | response to drug | 5.74E-13 | 13 | IL10, JUN, SRC, STAT3, PTGS2, ICAM1, IL4, IL6, IFNG, CASP3, CAT, CTNNB1, PPARG |
| 5 |  | GO:0045893 | positive regulation of transcription, DNA-templated | 7.03E-13 | 15 | IL10, JUN, SRC, EGF, STAT3, HIF1A, ESR1, TNF, IL4, IL6, IL1B, CTNNB1, PPARG, TP53, MAPK3 |
| 6 |  | GO:0051091 | positive regulation of sequence-specific DNA binding transcription factor activity | 3.65E-11 | 9 | IL10, IL4, IL6, IL1B, CTNNB1, AKT1, PPARG, ESR1, TNF |
| 7 |  | GO:0031663 | lipopolysaccharide-mediated signaling pathway | 4.55E-11 | 7 | NOS3, IL1B, CCL2, AKT1, TNF, TLR4, MAPK3 |
| 8 |  | GO:0070374 | positive regulation of ERK1 and ERK2 cascade | 6.60E-11 | 10 | IL6, JUN, SRC, CCL2, TNF, TLR4, EGFR, ICAM1, MAPK3, VEGFA |
| 9 |  | GO:0071222 | cellular response to lipopolysaccharide | 6.62E-11 | 9 | IL10, IL6, CXCL8, IFNG, SRC, CCL2, TNF, TLR4, ICAM1 |
| 10 |  | GO:0001525 | angiogenesis | 5.72E-10 | 10 | JUN, CXCL8, EGF, NOS3, LEP, CCL2, PTGS2, HIF1A, SIRT1, VEGFA |
| 11 | Molecular Function | GO:0019899 | enzyme binding | 4.08E-11 | 12 | JUN, SRC, CAT, CTNNB1, AKT1, PPARG, PTGS2, HIF1A, SIRT1, ESR1, TP53, EGFR |
| 12 |  | GO:0042802 | identical protein binding | 9.90E-11 | 15 | JUN, STAT3, SIRT1, ESR1, TNF, MMP9, EGFR, ACTB, INS, VEGFA, ALB, AKT1, PPARG, APOE, TP53 |
| 13 |  | GO:0005125 | cytokine activity | 1.49E-06 | 7 | IL10, IL4, IL6, IFNG, IL1B, TNF, VEGFA |
| 14 |  | GO:0008134 | transcription factor binding | 1.59E-06 | 8 | JUN, STAT3, CTNNB1, PPARG, HIF1A, SIRT1, ESR1, TP53 |
| 15 |  | GO:0019903 | protein phosphatase binding | 8.45E-06 | 5 | STAT3, CTNNB1, PPARG, TP53, EGFR |
| 16 |  | GO:0008083 | growth factor activity | 1.97E-05 | 6 | IL10, IL4, IL6, EGF, LEP, VEGFA |
| 17 |  | GO:0044212 | transcription regulatory region DNA binding | 7.28E-05 | 6 | JUN, STAT3, CTNNB1, PPARG, TNF, TP53 |
| 18 |  | GO:0001046 | core promoter sequence-specific DNA binding | 9.52E-05 | 4 | PPARG, SIRT1, ESR1, TP53 |
| 19 |  | GO:0030235 | nitric-oxide synthase regulator activity | 1.16E-04 | 3 | AKT1, ESR1, EGFR |
| 20 |  | GO:0005515 | protein binding | 1.46E-04 | 30 | CXCL8, SRC, PTGS2, HIF1A, TNF, EGFR, ACTB, INS, ICAM1, CASP3, AKT1, APOE, MAPK3, IL10, JUN, EGF, NOS3, ANXA5, STAT3, MMP9, SIRT1, ESR1, VEGFA, IL4, IL6, ALB, CTNNB1, PPARG, TP53, TLR4 |
| 21 | Cellular Component | GO:0005615 | extracellular space | 3.67E-13 | 20 | IL10, VCAM1, CXCL8, EGF, TNF, MMP9, EGFR, ACTB, INS, ICAM1, VEGFA, IL4, IL6, IFNG, IL1B, LEP, CAT, ALB, CCL2, APOE |
| 22 |  | GO:0009897 | external side of plasma membrane | 1.38E-07 | 8 | IL4, IL6, VCAM1, IFNG, ANXA5, TNF, TLR4, ICAM1 |
| 23 |  | GO:0005576 | extracellular region | 6.52E-07 | 15 | IL10, CXCL8, EGF, TNF, MMP9, INS, VEGFA, IL4, IL6, IFNG, IL1B, LEP, ALB, CCL2, APOE |
| 24 |  | GO:0005925 | focal adhesion | 9.00E-05 | 7 | ANXA5, CAT, CTNNB1, EGFR, ACTB, ICAM1, MAPK3 |
| 25 |  | GO:0043234 | protein complex | 1.20E-04 | 7 | ALB, CTNNB1, AKT1, PTGS2, TP53, ACTB, MAPK3 |
| 26 |  | GO:0005901 | caveola | 2.61E-04 | 4 | SRC, NOS3, PTGS2, MAPK3 |
| 27 |  | GO:0005737 | cytoplasm | 3.90E-04 | 21 | IL10, NOS3, SRC, ANXA5, STAT3, PTGS2, HIF1A, SIRT1, ESR1, EGFR, ACTB, VEGFA, IL6, IFNG, CASP3, LEP, AKT1, CTNNB1, APOE, TP53, TLR4 |
| 28 |  | GO:0000790 | nuclear chromatin | 4.94E-04 | 5 | STAT3, SIRT1, ESR1, TP53, ACTB |
| 29 |  | GO:0005719 | nuclear euchromatin | 0.00122 | 3 | JUN, CTNNB1, SIRT1 |
| 30 |  | GO:0090575 | RNA polymerase II transcription factor complex | 0.001823 | 3 | STAT3, PPARG, HIF1A |

**Table S3**. TSMT alleviates TOP 20 KEGG pathway of core targets of atherosclerosis

| NO. | ID | Name | p Value | Count | Genes |
| --- | --- | --- | --- | --- | --- |
| 1 | hsa05417 | Lipid and atherosclerosis | 6.38E-20 | 18 | AKT1, ICAM1, IL1B, IL6, CXCL8, JUN, MMP9, NOS3, PPARG, MAPK3, CCL2, SRC, STAT3, TLR4, TNF, TP53, VCAM1, CASP3 |
| 2 | hsa04933 | AGE-RAGE signaling pathway in diabetic complications | 1.40E-18 | 14 | AKT1, ICAM1, IL1B, IL6, CXCL8, JUN, NOS3, MAPK3, CCL2, STAT3, TNF, VCAM1, VEGFA, CASP3 |
| 3 | hsa05418 | Fluid shear stress and atherosclerosis | 3.70E-18 | 15 | CTNNB1, AKT1, ICAM1, IFNG, IL1B, JUN, MMP9, NOS3, ACTB, CCL2, SRC, TNF, TP53, VCAM1, VEGFA |
| 4 | hsa05205 | Proteoglycans in cancer | 1.39E-15 | 15 | CTNNB1, EGFR, AKT1, ESR1, HIF1A, MMP9, MAPK3, ACTB, SRC, STAT3, TLR4, TNF, TP53, VEGFA, CASP3 |
| 5 | hsa04657 | IL-17 signaling pathway | 1.94E-15 | 12 | IFNG, IL1B, IL4, IL6, CXCL8, JUN, MMP9, MAPK3, PTGS2, CCL2, TNF, CASP3 |
| 6 | hsa05144 | Malaria | 5.05E-15 | 10 | ICAM1, IFNG, IL1B, IL6, CXCL8, IL10, CCL2, TLR4, TNF, VCAM1 |
| 7 | hsa05163 | Human cytomegalovirus infection | 5.63E-15 | 15 | CTNNB1, EGFR, AKT1, IL1B, IL6, CXCL8, MAPK3, PTGS2, CCL2, SRC, STAT3, TNF, TP53, VEGFA, CASP3 |
| 8 | hsa04066 | HIF-1 signaling pathway | 1.22E-14 | 12 | EGF, EGFR, AKT1, HIF1A, IFNG, IL6, INS, NOS3, MAPK3, STAT3, TLR4, VEGFA |
| 9 | hsa04668 | TNF signaling pathway | 1.71E-14 | 12 | AKT1, ICAM1, IL1B, IL6, JUN, MMP9, MAPK3, PTGS2, CCL2, TNF, VCAM1, CASP3 |
| 10 | hsa05167 | Kaposi sarcoma-associated herpesvirus infection | 1.91E-14 | 14 | CTNNB1, AKT1, HIF1A, ICAM1, IL6, CXCL8, JUN, MAPK3, PTGS2, SRC, STAT3, TP53, VEGFA, CASP3 |
| 11 | hsa05135 | Yersinia infection | 2.01E-13 | 12 | AKT1, IL1B, IL6, CXCL8, IL10, JUN, MAPK3, ACTB, CCL2, SRC, TLR4, TNF |
| 12 | hsa05142 | Chagas disease | 2.36E-13 | 11 | AKT1, IFNG, IL1B, IL6, CXCL8, IL10, JUN, MAPK3, CCL2, TLR4, TNF |
| 13 | hsa05161 | Hepatitis B | 1.52E-12 | 12 | AKT1, IL6, CXCL8, JUN, MMP9, MAPK3, SRC, STAT3, TLR4, TNF, TP53, CASP3 |
| 14 | hsa05164 | Influenza A | 2.91E-12 | 12 | AKT1, CAM1, IFNG, IL1B, IL6, CXCL8, MAPK3, ACTB, CCL2, TLR4, TNF, CASP3 |
| 15 | hsa05323 | Rheumatoid arthritis | 3.52E-12 | 10 | ICAM1, IFNG, IL1B, IL6, CXCL8, JUN, CCL2, TLR4, TNF, VEGFA |
| 16 | hsa05219 | Bladder cancer | 4.45E-12 | 8 | EGF, EGFR, CXCL8, MMP9, MAPK3, SRC, TP53, VEGFA |
| 17 | hsa05321 | Inflammatory bowel disease | 4.65E-12 | 9 | IFNG, IL1B, IL4, IL6, IL10, JUN, STAT3, TLR4, TNF |
| 18 | hsa05133 | Pertussis | 2.01E-11 | 9 | IL1B, IL6, CXCL8, IL10, JUN, MAPK3, TLR4, TNF, CASP3 |
| 19 | hsa05140 | Leishmaniasis | 2.27E-11 | 9 | IFNG, IL1B, IL4, IL10, JUN, MAPK3, PTGS2, TLR4, TNF |
| 20 | hsa05235 | PD-L1 expression and PD-1 checkpoint pathway in cancer | 8.63E-11 | 9 | EGF, EGFR, AKT1, HIF1A, IFNG, JUN, MAPK3, STAT3, TLR4 |

**Table S4**. Crucial anti-AS ingredients were selected as candidate active compounds in TSMT

| NO. | name | Degree | Betweenness Centrality | Closeness Centrality | Category |
| --- | --- | --- | --- | --- | --- |
| 1 | Ferulic acid | 15 | 0.011623 | 0.439425 | compound |
| 2 | Genistin | 12 | 0.009253 | 0.468271 | compound |
| 3 | Glycyrrhizic acid | 12 | 0.008825 | 0.450526 | compound |
| 4 | 18-*β*-Glycyrrhetinic acid | 11 | 0.008666 | 0.470330 | compound |
| 5 | Luteolin | 10 | 0.007750 | 0.452431 | compound |
| 6 | Formononetin | 10 | 0.005829 | 0.420432 | compound |
| 7 | *α*-Linolenic acid | 9 | 0.010613 | 0.450526 | compound |
| 8 | Liquiritigenin | 8 | 0.002910 | 0.354892 | compound |
| 9 | *ρ*-Coumaric acid | 7 | 0.002227 | 0.373473 | compound |
| 10 | Senkyunolide C | 6 | 0.002210 | 0.398510 | compound |
| 11 | Pinocembrin | 6 | 0.001660 | 0.359664 | compound |
| 12 | Liquiritin | 5 | 0.001314 | 0.363328 | compound |
| 13 | Neoisoliquiritin | 5 | 0.001132 | 0.340223 | compound |
| 14 | Atractylenolide II | 5 | 0.001996 | 0.353719 | compound |
| 15 | Quinic acid | 4 | 0.001294 | 0.378761 | compound |
| 16 | Loganin | 4 | 0.000998 | 0.351396 | compound |
| 17 | 3-Hexenyl-beta-D-glucopyranoside | 4 | 0.000786 | 0.343499 | compound |
| 18 | Senkyunolide I | 4 | 0.000475 | 0.327718 | compound |
| 19 | Kingiside | 3 | 0.000521 | 0.332815 | compound |
| 20 | Senkyunolide D | 3 | 0.000475 | 0.327718 | compound |
| 21 | 5-Hydroxymethylfurfural | 3 | 0.000385 | 0.320840 | compound |
| 22 | Butylphthalide | 3 | 0.000555 | 0.292750 | compound |
| 23 | Nicotiflorin | 3 | 0.000779 | 0.347967 | compound |
| 24 | Achyranthine | 2 | 0.000183 | 0.313324 | compound |
| 25 | Loganic acid | 2 | 0.000183 | 0.313324 | compound |
| 26 | Vicenin-2 | 2 | 0.000272 | 0.318927 | compound |
| 27 | Sweroside | 2 | 0.000275 | 0.318927 | compound |
| 28 | Naringenin 7-O-glucoside | 2 | 0.000051 | 0.286479 | compound |
| 29 | 9,12,13-trihydroxy-10-octadecenoic acid | 2 | 0.000085 | 0.286479 | compound |
| 30 | MAPK3 | 102 | 0.187263 | 0.529703 | target |
| 31 | AKT1 | 96 | 0.167519 | 0.514423 | target |
| 32 | TNF | 72 | 0.105946 | 0.461207 | target |
| 33 | JUN | 56 | 0.064911 | 0.429719 | target |
| 34 | IL6 | 53 | 0.051269 | 0.426295 | target |
| 35 | EGFR | 53 | 0.051306 | 0.422925 | target |
| 36 | TP53 | 52 | 0.059002 | 0.421260 | target |
| 37 | EGF | 51 | 0.031663 | 0.419608 | target |
| 38 | CASP3 | 50 | 0.060152 | 0.421260 | target |
| 39 | IL1B | 44 | 0.029994 | 0.409962 | target |
| 40 | SRC | 42 | 0.035951 | 0.402256 | target |
| 41 | STAT3 | 35 | 0.021784 | 0.396296 | target |
| 42 | CXCL8 | 34 | 0.018404 | 0.394834 | target |
| 43 | TLR4 | 33 | 0.015619 | 0.391941 | target |
| 44 | IFNG | 30 | 0.014303 | 0.386282 | target |
| 45 | PTGS2 | 28 | 0.025922 | 0.387681 | target |
| 46 | VEGFA | 28 | 0.012774 | 0.383513 | target |
| 47 | CTNNB1 | 28 | 0.017519 | 0.382143 | target |
| 48 | INS | 28 | 0.018215 | 0.383513 | target |
| 49 | ACTB | 24 | 0.015287 | 0.376761 | target |
| 50 | IL10 | 22 | 0.013939 | 0.372822 | target |
| 51 | NOS3 | 21 | 0.009457 | 0.375439 | target |
| 52 | ESR1 | 21 | 0.014253 | 0.372822 | target |
| 53 | MMP9 | 21 | 0.009781 | 0.375439 | target |
| 54 | CCL2 | 19 | 0.004004 | 0.368966 | target |
| 55 | PPARG | 17 | 0.011288 | 0.370242 | target |
| 56 | IL4 | 16 | 0.008146 | 0.357860 | target |
| 57 | ICAM1 | 15 | 0.007887 | 0.361486 | target |
| 58 | HIF1A | 14 | 0.003429 | 0.362712 | target |
| 59 | VCAM1 | 10 | 0.003979 | 0.356667 | target |
| 60 | SIRT1 | 9 | 0.002453 | 0.351974 | target |
| 61 | CAT | 10 | 0.002665 | 0.359060 | target |
| 62 | LEP | 6 | 0.000749 | 0.340764 | target |
| 63 | APOE | 2 | 0.000024 | 0.326220 | target |

**Table S5**. Methodological investigation results of the content determination method of TSMT

| Reference Substance | Linearity | Range of Linearity (mg/L) | RSD% of Accuracy | RSD% of Repeatability | RSD% of Stability | Average Recoveries (%) | RSD% of Average Recoveries |
| --- | --- | --- | --- | --- | --- | --- | --- |
| Liquiritin | *Y* = 0.3227 *X* - 0.3177（*r*= 0.9998） | 2.64375~84.60 | 0.39 | 0.63 | 0.38 | 98.9 | 0.7 |
| Ferulic acid | Y= 0.5855 *X* - 2.5933 （*r*= 0.9998） | 11.25~360 | 0.19 | 2.46 | 1.4 | 99.4 | 1.3 |
| Senkyunolide I | *Y* = 0.1319 *X* - 0.2106 （*r*= 0.9998） | 4.1375~132.40 | 0.22 | 0.98 | 2.84 | 99.7 | 0.8 |
| Luteolin | *Y* = 0.4712 *X* - 0.8213 （*r*= 0.9997） | 3.5125~112.40 | 0.17 | 1.38 | 1.17 | 99.4 | 1.2 |
| Glycyrrhizic acid | *Y* = 0.0689 *X* - 0.2492（*r*= 0.9997） | 6.925~221.60 | 0.17 | 0.06 | 0.14 | 99.5 | 0.8 |

**Table S6**. Similarity evaluation results of HPLC fingerprint of nine batches of S1-S9 in TSMT

| NO. | S1 | S2 | S3 | S4 | S5 | S6 | S7 | S8 | S9 | R |
| --- | --- | --- | --- | --- | --- | --- | --- | --- | --- | --- |
| S1 | 1.000 | 1.000 | 0.999 | 0.998 | 0.999 | 0.999 | 0.999 | 0.998 | 0.999 | 0.999 |
| S2 | 1.000 | 1.000 | 0.999 | 0.998 | 0.999 | 0.999 | 0.999 | 0.998 | 0.999 | 0.999 |
| S3 | 0.999 | 0.999 | 1.000 | 0.999 | 0.999 | 1.000 | 1.000 | 1.000 | 0.999 | 1.000 |
| S4 | 0.998 | 0.998 | 0.999 | 1.000 | 0.999 | 0.999 | 0.999 | 0.999 | 0.998 | 0.999 |
| S5 | 0.999 | 0.999 | 0.999 | 0.999 | 1.000 | 0.999 | 0.999 | 1.000 | 1.000 | 1.000 |
| S6 | 0.999 | 0.999 | 1.000 | 0.999 | 0.999 | 1.000 | 1.000 | 1.000 | 0.999 | 1.000 |
| S7 | 0.999 | 0.999 | 1.000 | 0.999 | 0.999 | 1.000 | 1.000 | 1.000 | 0.999 | 1.000 |
| S8 | 0.998 | 0.998 | 1.000 | 0.999 | 1.000 | 1.000 | 1.000 | 1.000 | 1.000 | 1.000 |
| S9 | 0.999 | 0.999 | 0.999 | 0.998 | 1.000 | 0.999 | 0.999 | 1.000 | 1.000 | 1.000 |
| R | 0.999 | 0.999 | 1.000 | 0.999 | 1.000 | 1.000 | 1.000 | 1.000 | 1.000 | 1.000 |
